# Chromosomal instability-induced senescence potentiates cell non-autonomous tumourigenic effects

**DOI:** 10.1038/s41389-018-0072-4

**Published:** 2018-08-15

**Authors:** Qianqian He, Bijin Au, Madhura Kulkarni, Yang Shen, Kah. J. Lim, Jiamila Maimaiti, Cheng. Kit. Wong, Monique. N. H. Luijten, Han C. Chong, Elaine H. Lim, Giulia Rancati, Indrajit Sinha, Zhiyan Fu, Xiaomeng Wang, John. E. Connolly, Karen C. Crasta

**Affiliations:** 1 Lee Kong Chian School of Medicine, Nanyang Technological University, Singapore, Singapore; 2 School of Biological Sciences, Nanyang Technological University, Singapore, Singapore; 3 Institute of Molecular and Cell Biology, Agency for Science, Technology and Research, Singapore, Singapore; 4 Genome Institute of Singapore, Agency for Science, Technology and Research, Singapore, Singapore; 5 Acenzia Inc., Windsor, Ontario, Canada; 6 Institute of Medical Biology, Agency for Science, Technology and Research, Singapore, Singapore; 7 Division of Medical Oncology, National Cancer Centre Singapore, Singapore, Singapore; 8 Yong Loo Lin School of Medicine, National University of Singapore, Singapore, Singapore; 9 Institute of Biomedical Studies, Baylor University, Waco, TX USA; 10 Department of Medicine, Imperial College London, London, UK; 11 Present Address: Transnational Cancer Research Centre: Prashanti Cancer Care Mission, Indian Institute of Science Education and Research, Pune, India

## Abstract

Chromosomal instability (CIN), a high rate of chromosome loss or gain, is often associated with poor prognosis and drug resistance in cancers. Aneuploid, including near-polyploid, cells contain an abnormal number of chromosomes and exhibit CIN. The post-mitotic cell fates following generation of different degrees of chromosome mis-segregation and aneuploidy are unclear. Here we used aneuploidy inducers, nocodazole and reversine, to create different levels of aneuploidy. A higher extent of aneuploid and near-polyploid cells in a given population led to senescence. This was in contrast to cells with relatively lower levels of abnormal ploidy that continued to proliferate. Our findings revealed that senescence was accompanied by DNA damage and robust p53 activation. These senescent cells acquired the senescence-associated secretory phenotype (SASP). Depletion of p53 reduced the number of senescent cells with concomitant increase in cells undergoing DNA replication. Characterisation of these SASP factors demonstrated that they conferred paracrine pro-tumourigenic effects such as invasion, migration and angiogenesis both in vitro and in vivo. Finally, a correlation between increased aneuploidy and senescence was observed at the invasive front in breast carcinomas. Our findings demonstrate functional non-equivalence of discernable aneuploidies on tumourigenesis and suggest a cell non-autonomous mechanism by which aneuploidy-induced senescent cells and SASP can affect the tumour microenvironment to promote tumour progression.

## Introduction

Most malignant tumours contain cells with numerical aneuploidy (whole-chromosome loss or gain). Indeed, almost ninety percent of solid tumours exhibit aneuploidy^1^, which has been associated with poor prognosis in many tumours^2–5^. Aneuploidy is frequently linked with chromosomal instability (CIN), a cellular state with propensity for chromosome mis-segregation resulting in high rates of whole-chromosome loss or gain^6^. CIN can be caused by defects in genes involved in the spindle assembly checkpoint (SAC), sister chromatid cohesion, kinetochore assembly and other processes that facilitate chromosome segregation^7,8^.

Mouse models of CIN gene mutations, particularly within SAC genes, have demonstrated that aneuploidy is not simply a by-product in tumorigenesis but is directly involved. CENP-E haploinsufficiency in mice caused aneuploidy and increased spontaneous tumour occurrence in spleen and lung tissues^9^, whereas mitotic delay by MAD2 overexpression promoted aneuploidy and widespread tumour occurrence^10^. In addition, mutations in SAC component BUB1B and centrosomal protein CEP57 caused mosaic variegated aneuploidy and hereditary cancers in humans^11,12^. Aneuploidy has also been shown to drive tumorigenesis by conferring quick adaptive advantages under selective conditions^13^. CIN can yield heterogeneous aneuploid tumour cell populations that increase metastasis and resistance to therapy^14,15^. In addition, chromosome copy number changes can modulate cancer driver genes and promote cancer genome evolution^16^. CIN and aneuploidy have also been described to potentiate structural abnormalities that lead to genomic instability^17,18^. Whole-chromosome mis-segregation and aneuploidy have been shown to yield structural lesions via micronuclei which can generate genomic instability^3,19^. Hence, there is an unequivocal link between aneuploidy and tumorigenesis.

Previous studies on transcriptional response to aneuploidy compared modal cell lines harbouring defined aneuploidy of specific chromosomes with diploid equivalents^20,21^. However, the majority of tumours are composed of cells with complex karyotypes (diverse chromosome assortment). Despite this finding, proven functions of random aneuploidies in promoting tumorigenesis are lacking. This prompted us to investigate the transcriptional response to heterogenous cell populations with discernible random aneuploidies.

Here we report downstream cell fate consequences and tumorigenic implications of cell populations with mild (cells with < 5 chromosomes lost or gained) and severe aneuploidy ( ≥ 5 chromosomes lost or gained, including polyploidy). Cells with severe aneuploidy entered senescence while mildly aneuploid cells continued to proliferate. Importantly, these senescent cells elicited the senescence-associated secretory phenotype (SASP) that engendered paracrine pro-tumourigenic effects. Interestingly, aneuploidy and senescence/SASP were observed predominantly at the invasive front in breast carcinomas. Our findings indicate that aneuploidy-induced senescence could represent a cell non-autonomous mechanism by which cancer cells with distinguishable random aneuploidies differentially promote tumorigenesis.

## Results

### Aneuploid cells display cell cycle- and stress-related changes in gene expression

**Fig. 1:**
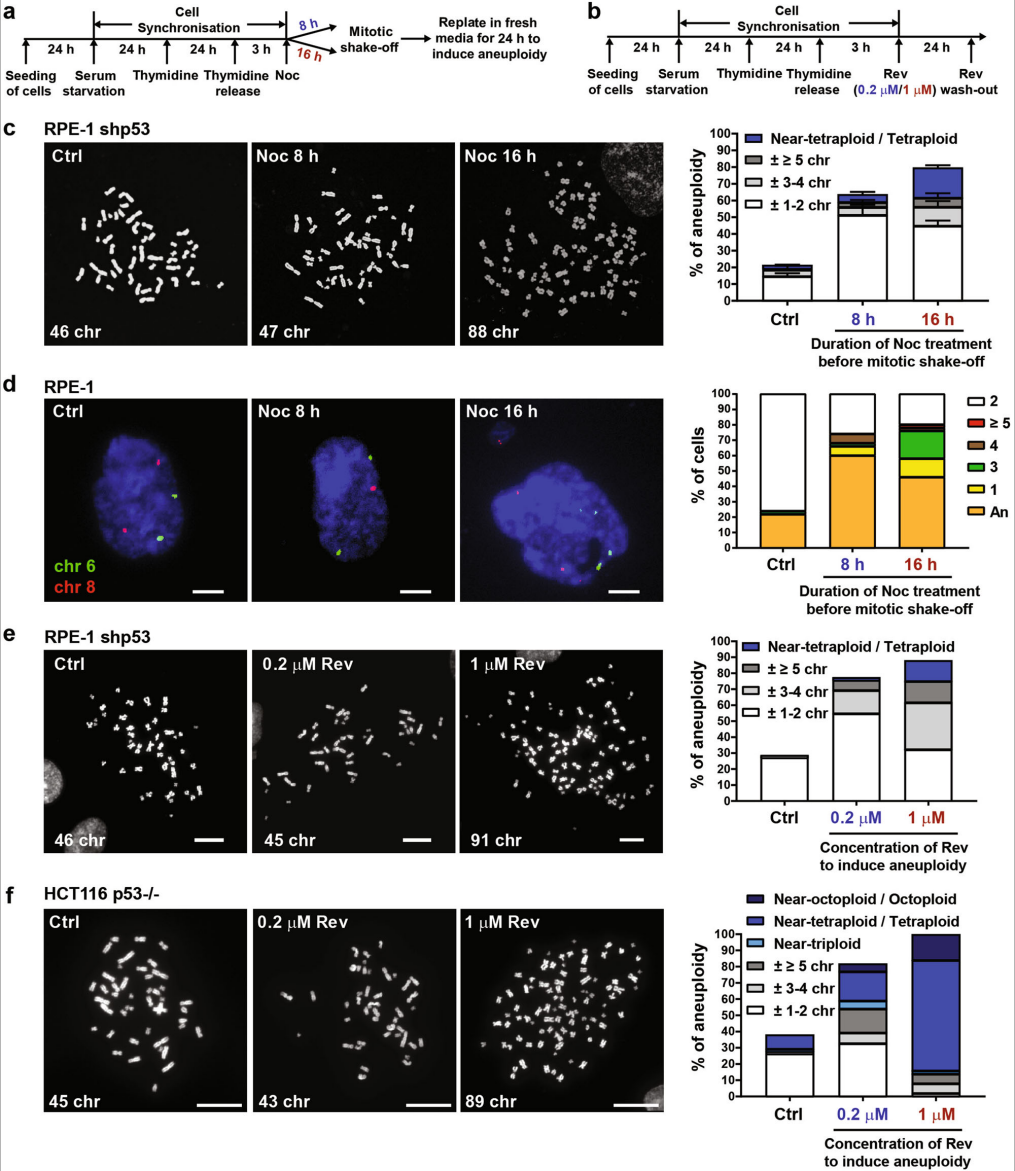
Nocodazole and reversine induce chromosome mis-segregation and aneuploidy in a duration- or dose-dependent manner. **a**, **b** Schematic representation of aneuploidy induction using either **(a)** 8 or 16 h nocodazole (Noc) or (**b)** 0.2 or 1 µM reversine (Rev). **c** Representative images of metaphase spreads from RPE-1 shp53 cells treated with Noc as per **a**. Plot shows percentage of cells with indicated number of chromosomes lost or gained. Data are presented as mean ± SD of two independent experiments. **d** Representative images of interphase FISH of RPE-1 cells treated with Noc as per **a** with probes for chromosome 6 (green) and chromosome 8 (red). Plot shows distribution of cell ploidy. ‘An’ represents nuclei with different numbers of signals for chromosomes 6 and 8. Scale bar, 10 µm. *n* = 50 cells. **e**, **f** Representative metaphase spreads from **(e)** RPE-1 shp53 cells and (**f**) HCT116 p53-null cells treated with Rev as per **b**. Plots show percentage of cells with indicated number of chromosomes gained or lost. Chr denotes chromosome. Near-triploidy: 60–80 chromosomes; near-tetraploidy/tetraploidy: 81–110 chromosomes; near-octoploidy/octoploidy: 170–182 chromosomes. Scale bar, 20 µm. Minimum of 50 spreads were analysed per condition

As a first step towards studying the impact of different degrees of aneuploidy on tumourigenesis, we induced aneuploidy in hTERT RPE-1 cells (non-transformed retinal pigment epithelial cells with modal chromosome number 46) and HCT116 cells (chromosomally stable colon cancer cells with modal chromosome number 45) via nocodazole (Noc) or reversine (Rev) treatments. These drugs promote chromosome mis-segregation and aneuploidy in distinct ways. The microtubule poison Noc induces merotely and lagging chromosomes after washout^22^, while Rev is a Mps1 inhibitor that overrides the SAC and accelerates mitotic progression even in the presence of improper kinetochore–microtubule attachments^23^. These were used either in a time- or dose-dependent manner, to generate different degrees of chromosome mis-segregation and aneuploidy (Noc 100 ng/ml for 8 h and 16 h; Rev 0.2 and 1 μM for 24 h) (Fig. 1a, b).

To better understand cell fate following Noc washout, RPE-1 cells were grown for an additional 24 h to generate aneuploidy after mitotic shake-off (Fig. 1a). Synchronised cells without drug treatment served as control. Time-lapse microscopy confirmed that mitotically arrested cells harboured mis-segregated chromosomes, defined as lagging chromosomes, broken chromatids and chromatin bridges (Supplementary Figures S1a-b). Notably, 16 h Noc treatment was observed to cause higher number of mis-segregated chromosomes per cell and cytokinetic failure compared with 8 h treatment. As mis-segregated chromosomes are often encapsulated in micronuclei, this could result in increased number of micronuclei (usually containing one o two chromosomes) or multinucleated cells (containing several chromosomes). Indeed, 16 h Noc treatment yielded increased micro- and multinucleation compared with 8 h treatment (Supplementary Figure S1c). Consistent with a previous report^24^, we observed a p53-dependent G1 cell cycle arrest following release from Noc arrest (Supplementary Figures S1d-e). To determine aneuploidy by metaphase spreads, p53 was depleted (shp53) to allow cells to proceed to mitosis (Supplementary Figure S1f). Metaphase spread analyses revealed karyotypic heterogeneity between the conditions, with 16 h Noc-treated cells displaying a greater extent of chromosome number loss or gain, and an increased near-tetraploid population, compared with their 8 h Noc-treated counterparts (Fig. 1c). Two-chromosomes interphase fluorescence in situ hybridisation (FISH) performed 24 h after release from Noc-induced arrest further confirmed this variation in aneuploidy (Fig. 1d).

Rev has been shown to accelerate progression through mitosis rather than enforce a mitotic arrest, with induction of chromosome mis-segregation occurring in a dose-dependent manner^23,25^. Indeed, 1 µM Rev treatment (24 h) induced a significant increase in chromosome mis-segregation compared to 0.2 µM Rev treatment (Supplementary Figures S2a-b). Moreover, 1 µM Rev also caused a significant increase in micronucleated and multinucleated cells (Supplementary Figure S2c). As Rev was also found to induce p53-dependent cell cycle arrest similar to Noc treatment (Supplementary Figures S2d-e), RPE-1 shp53 cells were used for metaphase spreads. Rev (1 and 0.2 µM) were used to induce comparatively ‘severe’ and ‘mild’ forms of aneuploidy, respectively. Metaphase spreads of cells collected immediately following treatment with 1 μM Rev confirmed an increase in overall aneuploidy and a preferential increase in severe form of aneuploidy ( ≥ 5 chromosomes lost or gained, including near-tetraploidy/tetraploidy (81–110 chromosomes)) compared with control cells (Fig. 1e). In contrast, cells treated with 0.2 μM Rev displayed an increase in cells with < 5 chromosomes lost or gained (Fig. 1e). To test whether Rev has a similar effect on chromosomally stable HCT116 cancer cells, we prepared metaphase spreads in Rev-treated HCT116 p53-null cells due to p53 activation in HCT116 wild-type (WT) cells (Supplementary Figure S2f). Rev (1 µM) treatment was found to increase polyploidy, including near-triploidy (60–80 chromosomes), near-tetraploidy/tetraploidy (81–110 chromosomes) and near-octoploidy/octoploidy (170–182 chromosomes), whereas 0.2 µM Rev induced relatively mild form of aneuploidy (Fig. 1f). Hereafter, we refer to 1 µM Rev and 16 h Noc-treated cells as severely aneuploid cell populations and their respective counterparts treated with 0.2 µM Rev and 8 h Noc as mildly aneuploid cell populations.

**Fig. 2:**
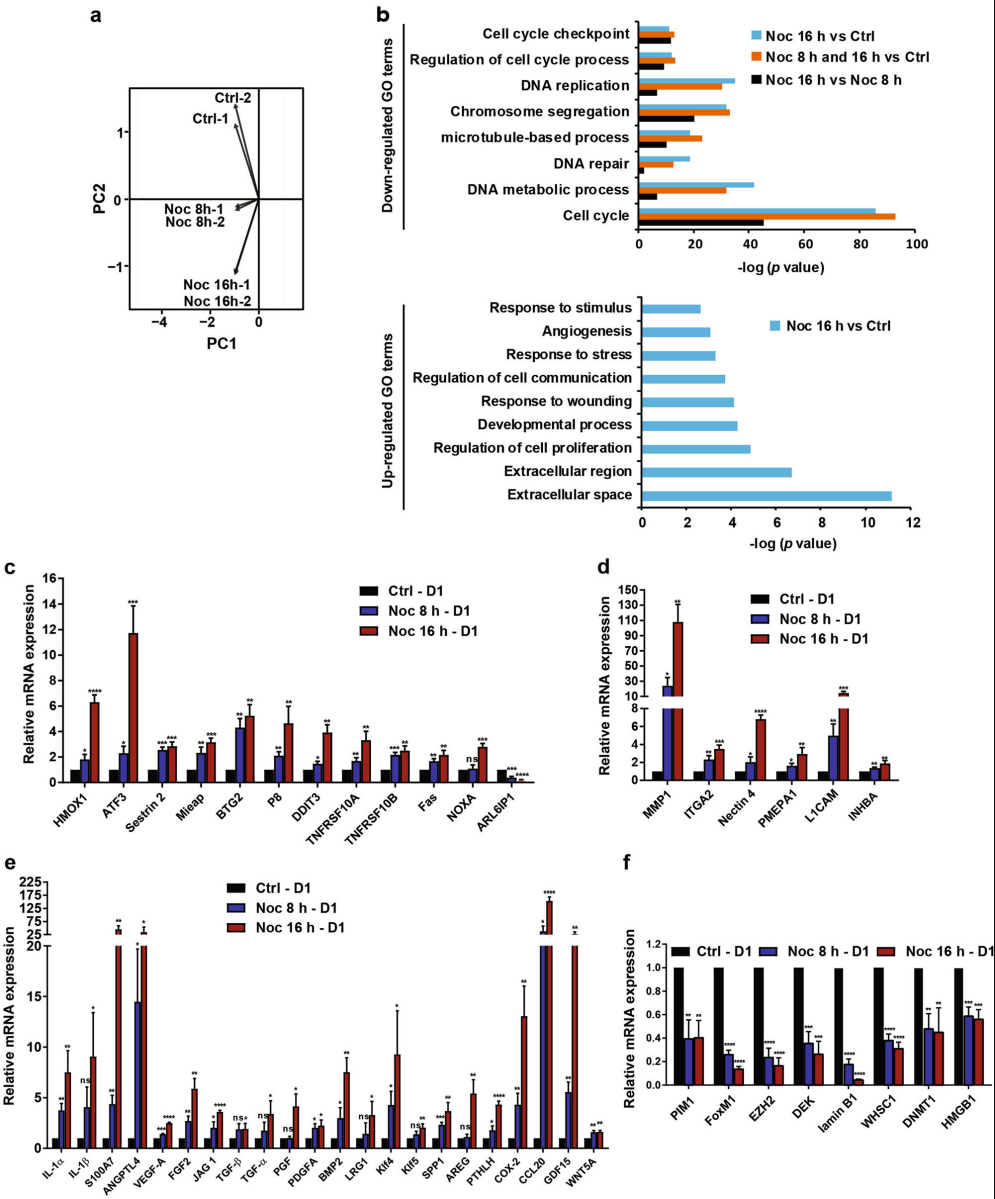
Aneuploidy causes cell cycle- and stress-related transcriptional changes. **a**, **b** RPE-1 cells were treated as per Fig. 1a and subjected to RNA-sequencing (RNA-seq) analysis. (**a)** PCA (principle component analysis) plot and (**b)** GO (Gene Ontology) analysis. Downregulated GO terms in (**b**) indicate enrichment within aneuploid cells induced by Noc 8 h and 16 h treatments. Upregulated GO terms in (**b**) depict enrichment within Noc 16 h-induced aneuploid cells. GO terms with Benjamini *P*-value < 0.05 were considered to be significant. The *x*-axis in (**b**) indicates − log (Benjamini *P*-value). *n* = 2. **c–f** Validation of hits from RNA-seq by qRT-PCR analysis. Plots show fold changes of mRNA expression of (**c**) stress-related genes, **(d)** migration-related genes, (**e**) pro-inflammatory factors, and **(f**) transcription and chromatin-remodelling factors. mRNA levels were normalised to GAPDH. D1 represents 1 day after mitotic shake-off. All data are expressed as mean ± SD of three independent experiments. *P < 0.05, **P < 0.01, ***P < 0.001, ****P < 0.0001. NS not significant by Student’s *t*-test

Based on our results above, aneuploid RPE-1 cell populations were collected 24 h after 8 or 16 h Noc washout treatments for differential gene expression analyses by RNA-sequencing (RNA-seq). Principal component analysis (PCA) demonstrated that gene expression patterns of the two biological replicates were highly correlative (Fig. 2a). (Full lists of upregulated and downregulated genes from RNA-seq data can be found in Supplementary Tables S1-S3. The data were filtered such that only genes that had twofold changes or greater over the control were shown). Gene Ontology (GO) analyses using DAVID^26^ revealed that downregulation of genes involved in cell cycle, DNA metabolic processes and DNA repair compared with control were common to both 8 and 16 h-treated cells (Noc 8 h and 16 h vs. Ctrl) (Fig. 2b top), with 16 h treatment (severely aneuploid) showing greater downregulation in these genes compared with 8 h treatment (Noc 16 h vs. Noc 8 h). Sixteen-hour treatment also displayed upregulation of genes associated with extracellular matrix, response to stimulus and stress, and response to wounding and angiogenesis compared with control (Fig. 2b bottom). It is noteworthy that gene expression changes observed with Noc-induced random aneuploidy shared high similarities with defined aneuploidy reported for both mouse and human cells^20,21^.

To validate the RNA-seq data, we performed quantitative real-time PCR (qRT-PCR) of genes of interest. Consistent with our RNA-seq data, qRT-PCR results also showed increased expression of genes associated with response to stress (Fig. 2c), cell migration (Fig. 2d), inflammatory response and angiogenesis (Fig. 2e), as well as downregulation of transcription and chromatin-remodelling factors (Fig. 2f) in severely aneuploid cells compared with control cells. The most obvious increase in gene expression in severely aneuploid cells compared with mildly aneuploid cells were those associated with the inflammatory response such as interleukin (IL)-1α, IL-1β, COX-2 and CCL20, as well as angiogenic factors such as VEGF-A, FGF2, LRG1, JAG 1, TGF-α, S100A7 and ANGPTL4 (Fig. 2e). Results from both RNA-seq and qRT-PCR results suggest that gene expression changes correlate with overall aneuploidy.

### Elevated chromosome mis-segregation and aneuploidy induce senescence and DNA damage

**Fig. 3:**
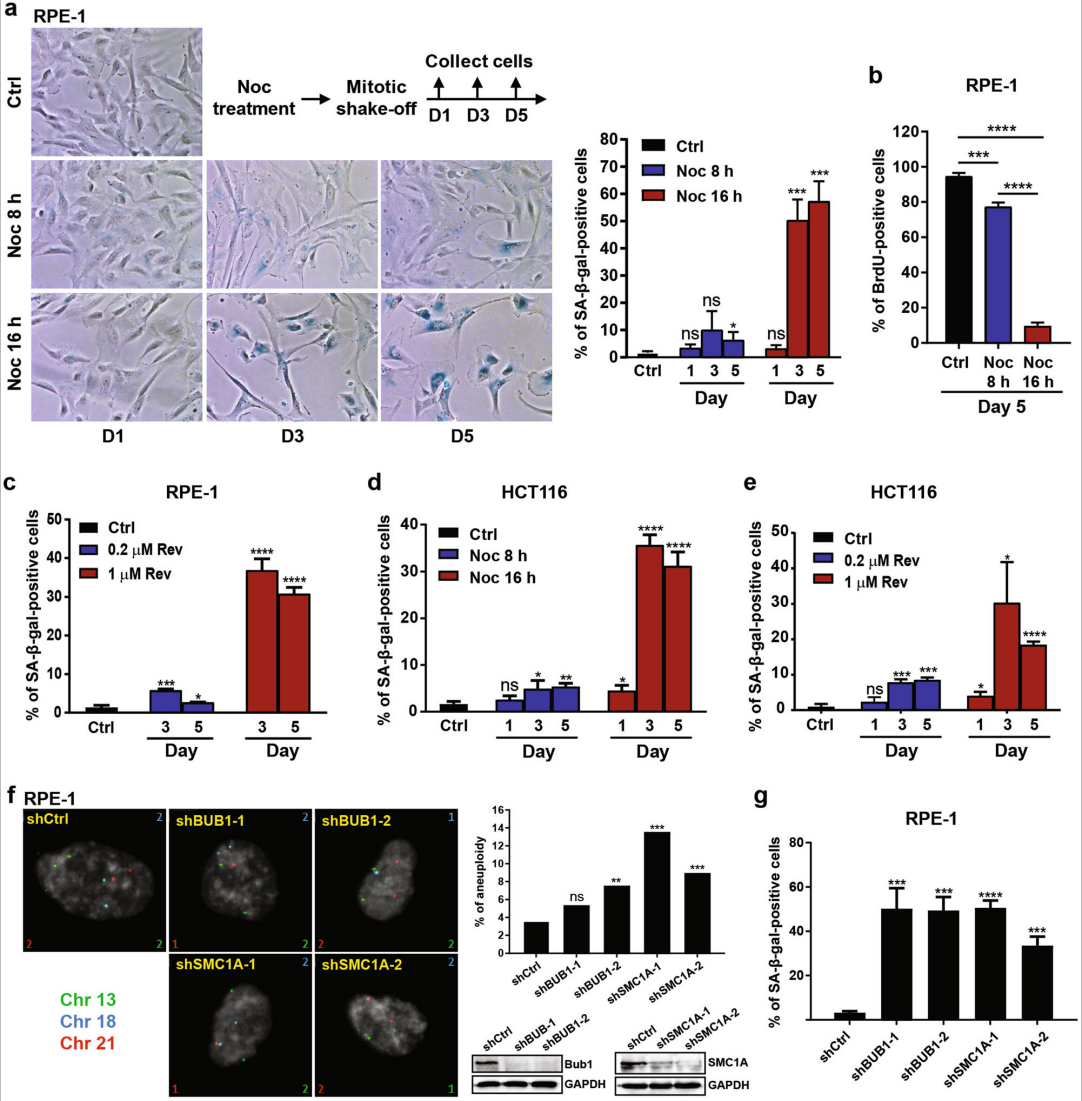
**Severely aneuploid cells enter senescence**. **a** RPE-1 cells were treated with Noc for 8 or 16 h, followed by mitotic shake-off and culture in drug-free media for indicated number of days. (Left) Representative bright-field images of cells stained for SA-β-gal. (Right) Plot shows percentage of SA-β-gal-positive cells. **b** RPE-1 cells were treated with Noc as per (**a**). BrdU incorporation assay was performed 5 days after mitotic shake-off. Plot shows percentage of BrdU-positive cells. **c** Percentage of SA-β-gal-positive RPE-1 cells after treatment with 0.2 µM or 1 µM Rev for 24 h and culture in drug-free media for indicated number of days. **d** Percentage of SA-β-gal-positive HCT116 cells treated with Noc for 8 or 16 h, followed by mitotic shake-off and culture in drug-free media for indicated number of days. **e** Percentage of SA-β-gal-positive HCT116 cells treated with 0.2 µM or 1 µM Rev for 24 h and cultured in drug-free media for indicated number of days. **f** (Left) Representative interphase FISH images of RPE-1 cells treated with indicated shRNA with probes for chromosome 13 (green), chromosome 18 (aqua) and chromosome 21 (red). Numbers at the corners of images indicate the number of chromosomes with the same colour. (Top right) Plot shows percentage of aneuploid cells based on chromosome 13, 18 and 21. *n* = 500 cells. **P < 0.01, ***P < 0.001. NS not significant by Fisher’s exact test. (Bottom right) Western blottings depicting knockdown efficiency of Bub1 and SMC1A. GAPDH was used as loading control. **g** Plot shows percentage of SA-β-gal-positive RPE-1 cells treated with indicated shRNA. Minimum of 100 cells were quantified per experimental condition in **a–e** and **g**, and data are expressed as mean ± SD of three independent experiments. *P*-values were derived from Student’s *t*-test (*P < 0.05, **P < 0.01, ***P < 0.001, ****P < 0.0001; NS not significant)

Our RNA-seq and qRT-PCR analyses clearly demonstrated that cells with highly aberrant karyotypes triggered a stress response (Fig. 2b, c). The stress response generally culminates in either cell cycle arrest, which may lead to senescence, or apoptotic cell death^27^. To determine cell fates following induction of aneuploidy, we collected RPE-1 cells from differentially treated populations at Day 1, 3 and 5 following mitotic shake-off. Intriguingly, cells collected 3 days following 16 h Noc treatment displayed an enlarged and flattened morphology reminiscent of senescence (a stable cell cycle arrest) (Fig. 3a). Indeed, a significant increase in senescence-associated β-galactosidase (SA-β-gal)-positive cells was observed at Day 3 in contrast to 8 h treatment where cells exhibited negligible increase in SA-β-gal activity (Fig. 3a). The senescence state was also confirmed by dramatic reduction in DNA synthesis as measured by 5-bromo-2′-deoxy-uridine (BrdU) incorporation (Fig. 3b). Similar results were also observed with the severely aneuploid population generated via Rev treatment (Fig. 3c). This was also found to be true in identically treated HCT116 cancer cells (Fig. 3d, e). These findings were further confirmed by generation of aneuploidy using genetic intervention mechanisms. Two short hairpin RNAs (shRNAs) against BUB1 and SMC1A were used to control for off-target effects. Suppression of either SAC component BUB1 or SMC1A cohesin subunit, which induced aneuploidy as assayed by three-chromosomes interphase FISH, increased percentage of SA-β-gal-positive RPE-1 cells (Fig. 3f, g). Taken together, these results demonstrate that cells with a higher degree of chromosome mis-segregation and aneuploidy could induce senescence in both normal and cancer cells.

As chromosome mis-segregation has been reported to cause DNA damage^18^, a well-known senescence stimulus, we asked whether DNA damage accompanied aneuploidy induction in our context. To this end, we used the well-characterised DNA damage marker γH2AX^28^. Immunofluorescence (IF) staining revealed increased γH2AX signal in Noc-induced severely aneuploid cells compared with mildly aneuploid counterparts and control (Supplementary Figure S3a). Consistent with this, immunoblots demonstrated greater DNA damage accumulation in severely aneuploid RPE-1 cells (Noc 16 h and 1 µM Rev) compared with mildly aneuploid cells (Noc 8 h and 0.2 µM Rev) and control (Supplementary Figures S3b-c). The increase in DNA damage correlated with elevated levels of senescence-associated markers p53 activation (phosphorylation on serine 15 residue of p53), p21 expression and lamin B1 loss. Notably, we did not observe significant difference in DNA damage in mildly aneuploid cells compared with the control. Rev (1 µM) also induced enhanced DNA damage and senescence-associated markers in HCT116 cells (Supplementary Figure S3d). Furthermore, knockdown of BUB1 and SMC1A also caused DNA damage and increased p21 expression in RPE-1 cells with the exception of shBUB1-1 (Supplementary Figure S3e). These results suggest that aneuploidy-associated stresses resulting in DNA damage correlate with severity of chromosome mis-segregation and degree of aneuploidy.

### Aneuploidy-induced senescence is dependent on p53

**Fig. 4:**
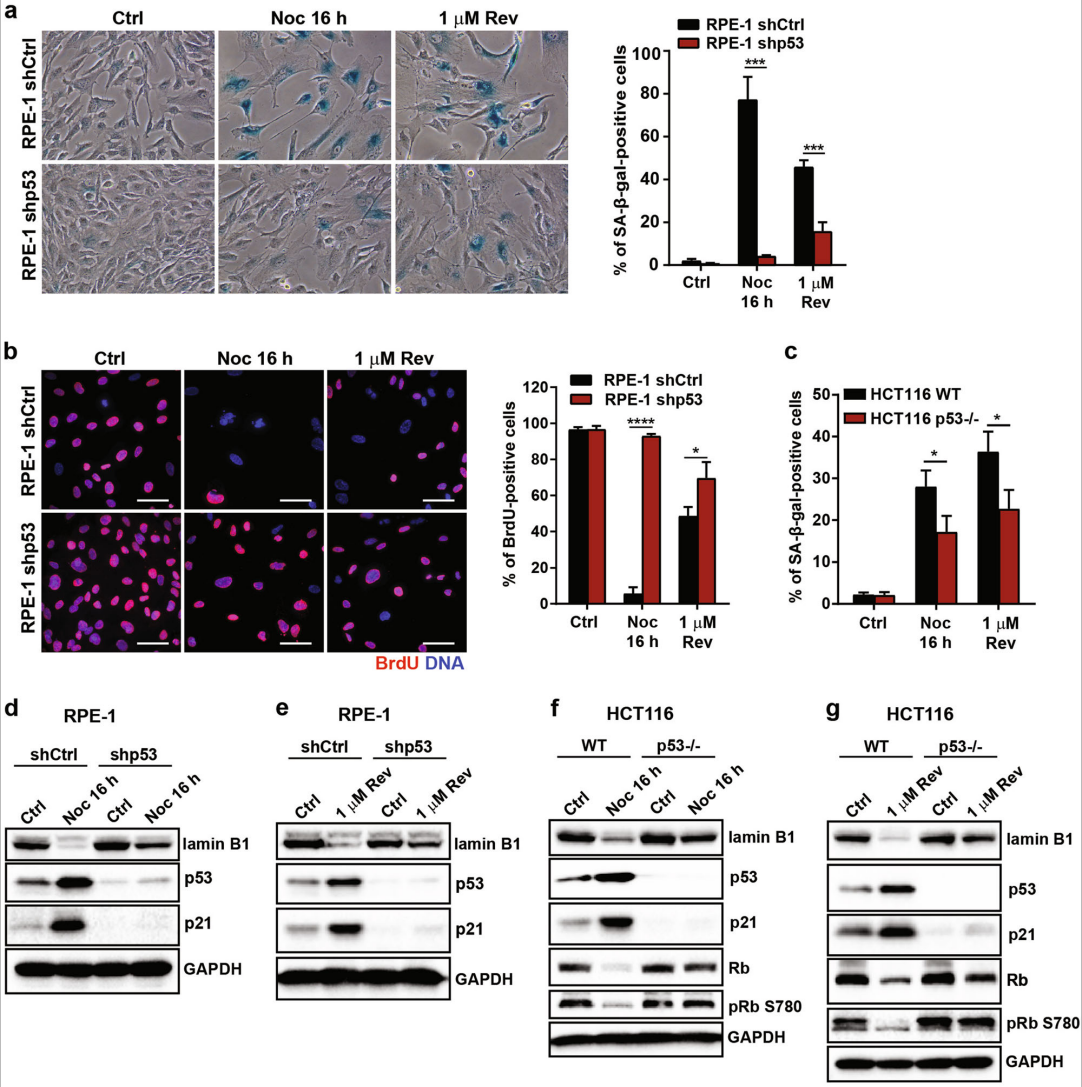
Aneuploidy-induced senescence is dependent on p53. **a**, **b** Representative images and plot showing percentage of **(a)** SA-β-gal-positive and **(b)** BrdU-positive RPE-1 shCtrl and shp53 cells treated with Noc for 16 h or 1 µM Rev for 24 h and cultured in drug-free media for 5 days. Scale bar, 50 µm. **c** Plot showing percentage of SA-β-gal-positive cells in HCT116 wide-type (WT) and p53-null cells treated with Noc for 16 h or 1 µM Rev for 24 h and cultured in drug-free media for 5 days. **d–g** Western blot analyses of indicated proteins in (**d**, **e**) RPE-1 shCtrl and shp53 cells or in (**f**, **g**) HCT116 WT and p53-null cells treated with Noc for 16 h or 1 µM Rev for 24 h and cultured for 5 days. GAPDH was used as loading control. Minimum of 100 cells were quantified per sample in all experiments. Data are expressed as mean ± SD of three independent experiments. All *P-*values were derived from Student’s *t*-test (*P < 0.05, ***P < 0.001, ****P < 0.0001)

We next asked whether p53, a canonical inducer of cellular senescence, contributed to aneuploidy-induced senescence. As shown in Supplementary Figures S3b-c, greater p53 stabilisation was observed in severely aneuploid cells in contrast to mildly aneuploid and control cells. Notably, consistent with previous studies describing downregulation of p53 activation following senescence induction^29^, we observed a decrease in p53 stabilisation and phosphorylation on serine 15 residue of p53 (Supplementary Figures S3b-d). As activation of p53 is known to be required for DNA damage- and oncogene-induced senescence^30,31^, we asked whether aneuploidy-induced senescence was also dependent on p53. To evaluate this, we depleted p53 in RPE-1 cells and tracked the cell fate of severely aneuploid cells induced via both Noc and Rev treatments. As shown in Fig. 4a, a dramatic decrease in SA-β-gal-positive cells at Day 5 was observed upon p53 depletion. Our findings were further substantiated by increased BrdU-labelling in p53-depleted RPE-1 cells (Fig. 4b). Similarly, HCT116 p53-null cells also showed significantly reduced SA-β-gal activity compared to WT upon both 16 h Noc and 1 µM Rev treatments (Fig. 4c). Consistently, p53 depletion in RPE-1 and HCT116 cells reduced p21 expression and increased lamin B1, Rb and phospho-Rb expression (Fig. 4d–g). Taken together, our results indicate that aneuploidy-induced senescence was dependent on p53.

### Aneuploidy-induced senescence displays pro-tumorigenic effects via its secretory phenotype

Senescent cells possess the potential to exhibit a complex secretome termed the SASP. The SASP consists of a wide range of biologically active factors, which include cytokines, chemokines, growth factors and matrix metalloproteinases that can affect the microenvironment^32^. Senescence is generally regarded as tumour suppressive, as cells undergo a stable cell cycle arrest. However, depending on the cell type and context, the SASP can be tumour suppressive (via autocrine and paracrine mechanisms)^33–36^ or pro-tumourigenic (via paracrine action)^37–41^.

**Fig. 5:**
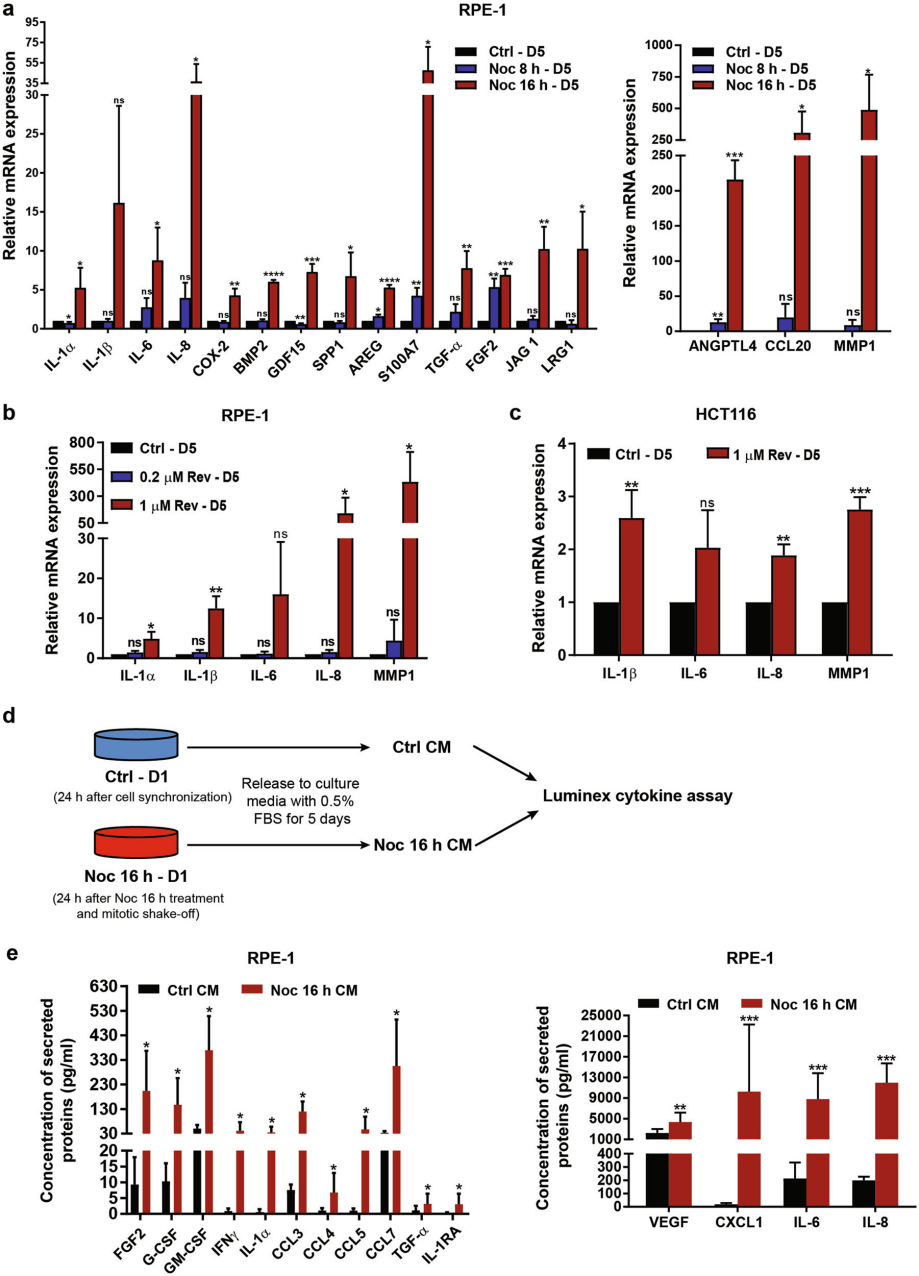
Aneuploidy-induced senescence promotes SASP. **a** qRT-PCR of SASP components and pro-tumourigenic factors in RPE-1 cells 5 days (D5) after mitotic shake-off from 8 h or 16 h Noc treatment. mRNA levels were normalised to actin. **b**, **c** qRT-PCR of SASP factors in (**b**) RPE-1 cells or **(c)** HCT116 cells 5 days (D5) following Rev washout. **d** Experimental schema of conditioned media (CM) preparation for Luminex cytokine assay. **e** Cytokine levels in CM of control and 16 h Noc-treated RPE-1 cells determined by Luminex cytokine assay. All data are expressed as mean ± SD from three independent experiments. *P < 0.05, **P < 0.01, ***P < 0.001, ****P < 0.0001. NS not significant by Student’s *t*-test

To assess whether aneuploidy-induced senescent cells develop SASP, we first examined mRNA expression of well-established SASP factors that included pro-inflammatory and growth-promoting factors such as IL-1α, IL-1β, IL-6, IL-8, FGF2, and MMP1. qRT-PCR analyses of severely aneuploid cells generated via both Noc and Rev, displayed significantly increased expression of these SASP-related genes compared with their mildly aneuploid counterparts and control cells (Fig. 5a–c). To quantitatively measure secreted SASP factors, we analysed conditioned media (CM) from Noc-induced severely aneuploid senescent cells by the Luminex assay (Fig. 5d). Various cytokines and chemokines such as IL-1α, IL-6, IL-8, VEGF, FGF2, CXCL1, CCL3, CCL4, CCL5 and CCL7 were observed to be significantly upregulated relative to control cells (Fig. 5e). These data suggest that aneuploidy-induced senescence is accompanied by SASP secretion. Interestingly, although loss of p53 inhibited senescence induction, qRT-PCR analyses of SASP factors such as IL-1α, IL-1β, IL-6, IL-8 and MMP1 in p53-depleted cells revealed enhanced mRNA expression in both RPE-1 and HCT116 cells (Supplementary Figures S4a-b). These findings are in agreement with a previous report from the Campisi lab that showed p53-depleted cells amplified SASP^37^.

**Fig. 6:**
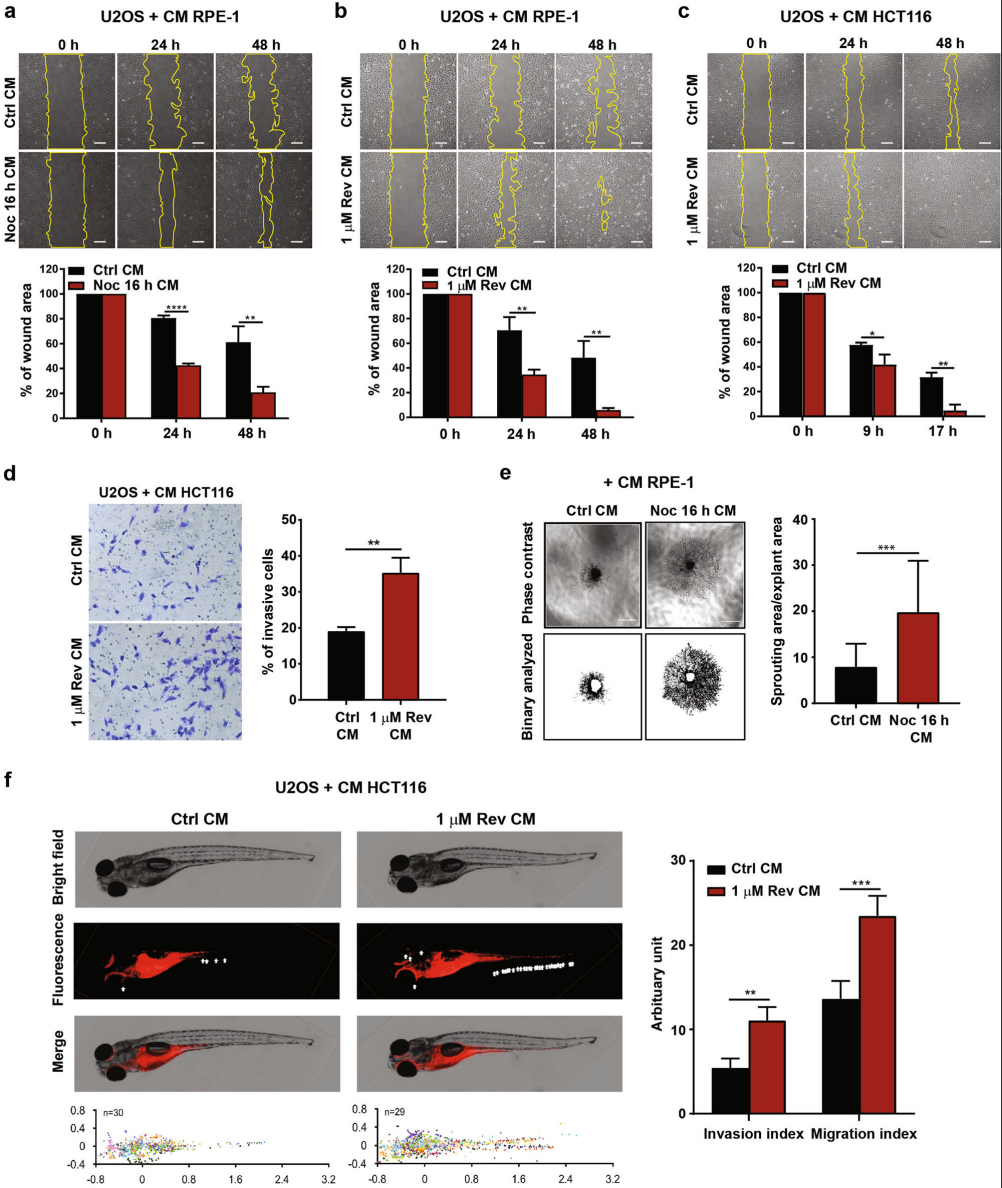
Aneuploidy-associated SASP facilitates paracrine tumourigenic phenotypes. **a**–**c** U2OS cells were incubated for 24 h with CM from **(a)** control or 16 h Noc-treated RPE-1 cells, (**b)** control or 1 µM Rev-treated RPE-1 cells, or (**c**) control or 1 µM Rev-treated HCT116 cells. Cells from the respective conditions were subsequently subjected to the scratch wound-healing assay. Representative phase-contrast images are shown. Plots show the percentage of wound area relative to the initial wound area at time 0 h (immediately after the scratch). All data are expressed as mean ± SD from three independent experiments. Scale bar, 200 µm. **d** Representative crystal violet stain images and plot of the percentage of invasive U2OS cells treated for 2 days with CM from control or 1 µM Rev-treated HCT116 cells, assessed by the transwell matrigel assay. **e** Representative images of choroid sprouts formed after incubation with CM from control or 16 h Noc-treated RPE-1 cells over 4 days. Plot shows ratio of choroid sprouting area to explant area. A minimum of six explants were analysed per experimental condition. All data are expressed as mean ± SD. *n* = 3. **f** U2OS cells were incubated with CM from control or 1 µM Rev-treated HCT116 cells over 2 days, stained using DiI and injected into zebrafish embryos. White arrows indicate U2OS cells that invaded the zebrafish body. All tumour foci observed in all tested larvae, belonging to each treatment group (presented as *n* on the graph), are presented as a scatter plot. Foci in one larva are represented by one colour. ‘n’ denotes number of injected embryos from three biological replicates. Metastatic ability upon different treatments was measured by Invasion index and Migration index. Data are presented as mean ± SEM from three independent experiments. Student’s *t*-test was used to evaluate significance between treatment groups: *P < 0.05, **P < 0.01, ***P < 0.001 and ****P < 0.0001

The SASP can evoke a pro-tumourigenic environment where SASP factors confer paracrine tumourigenic effects on neighbouring pre-malignant cells. To evaluate whether SASP factors from the aneuploidy-induced senescent cells have tumour-promoting capabilities, we utilised CM from RPE-1 and HCT116 cells for use in various phenotypic assays. To assess migration potential in vitro, the scratch wounding assay was used. Here, human osteosarcoma U2OS cells (chosen due to their metastatic potential) were exposed to CM from severely aneuploid senescent RPE-1 and HCT116 cells. As shown in Fig. 6a–c, CM from both aneuploidy-induced senescent RPE-1 and HCT116 cells promoted wound closure at a rate faster than control CM, indicating increased cell migration. Notably, the cell number and viability of U2OS cells were not significantly affected upon incubation with senescent CM compared to control CM (Supplementary Figures S5a-b). This ruled out any possible influence by cell proliferation and viability on the ability of aneuploidy-induced SASP to promote cell migration. In addition, to exclude the possibility that the above-mentioned paracrine effects were due to side-effects of drugs utilised, we assayed SASP factor expression and associated migratory properties following shSMC1A-induced senescence in RPE-1 cells. Our results demonstrated the upregulation of IL-8, MMP1, CCL20 and FGF2 compared with control (Supplementary Figure S6a). CM derived from these cells led to faster rate of wound closure, indicating that secretory factors from shSMC1A-induced senescence could also promote migratory capabilities of U2OS cells (Supplementary Figure S^6^b).

To test whether CM from aneuploidy-induced senescent cells promote cell invasion, we performed the transwell matrigel assay. Results showed that a higher number of cells exposed to senescent CM (1 µM Rev CM) invaded the bottom of the chamber than cells exposed to control CM, confirming the pro-invasive ability of SASP factors (Fig. 6d).

To assess pro-angiogenic capabilities, the ex vivo choroid angiogenesis assay was used. Here, mice choroid explants were incubated with CM derived from either severely aneuploid senescent cells (Noc 16 h) or control cells for 4 days. Choroid explants exposed to CM from severely aneuploid senescent cells showed increased vascular sprouting than that with control CM (Fig. 6e), indicating enhanced pro-angiogenic capability of SASP factors from the former. This was consistent with our RNA-seq (Fig. 2b) and qRT-PCR data (Figs. 2e and 5a) which showed upregulation of pro-angiogenic factors such as LRG1, ANGPTL4, VEGF and FGF2, along with other cytokines and chemokines.

To further investigate whether the observed in vitro migratory phenotype could be recapitulated in vivo, we used the zebrafish embryo model to monitor migration and invasion. The zebrafish embryo serves as a tractable animal model to evaluate metastasis of tumour cells due to its transparent feature, offering unique in vivo imaging possibilities. U2OS and HCT116 cells were incubated with either senescent or control CM for 2 days before injection into zebrafish embryos. For consistent quantitative comparison of differentially treated cells, we defined invasion index as the number of metastases formed per hundred cells injected, and migration index as the distance travelled by metastases as percent of the maximum distance cells can migrate within the zebrafish axis. U2OS and HCT116 cells incubated with CM from severely aneuploid senescent HCT116 cells were observed to migrate and invade farther from the injection site compared with control CM (Fig. 6f and Supplementary Figure S7a). Senescent CM from RPE-1 cells also promoted migration of U2OS cells from the injection site through the zebrafish body (i.e., invasion index) (Supplementary Figure S7b). However, these invasive cells did not show significant difference in the migration index (distance travelled by cell). This led us to speculate that senescent CM from normal cells like RPE-1 confer lower invasiveness compared with HCT116 cancer cells. Importantly, although senescent CM from HCT116 cells slightly increased U2OS cell proliferation compared with control CM from Day 3 (Supplementary Figure S7c), senescent CM generated from RPE-1 and HCT116 cells exerted no significant effect on cell proliferation on U2OS and HCT116 cells than control CM (Supplementary Figures S7d-e). This ruled out the possibility of cell proliferation affecting cell invasion in zebrafish embryos. Taken together, our findings support paracrine pro-tumourigenic potential of aneuploidy-associated SASP in vivo.

### Increased aneuploidy correlates with senescence at the invasive front in breast tumours

**Fig. 7:**
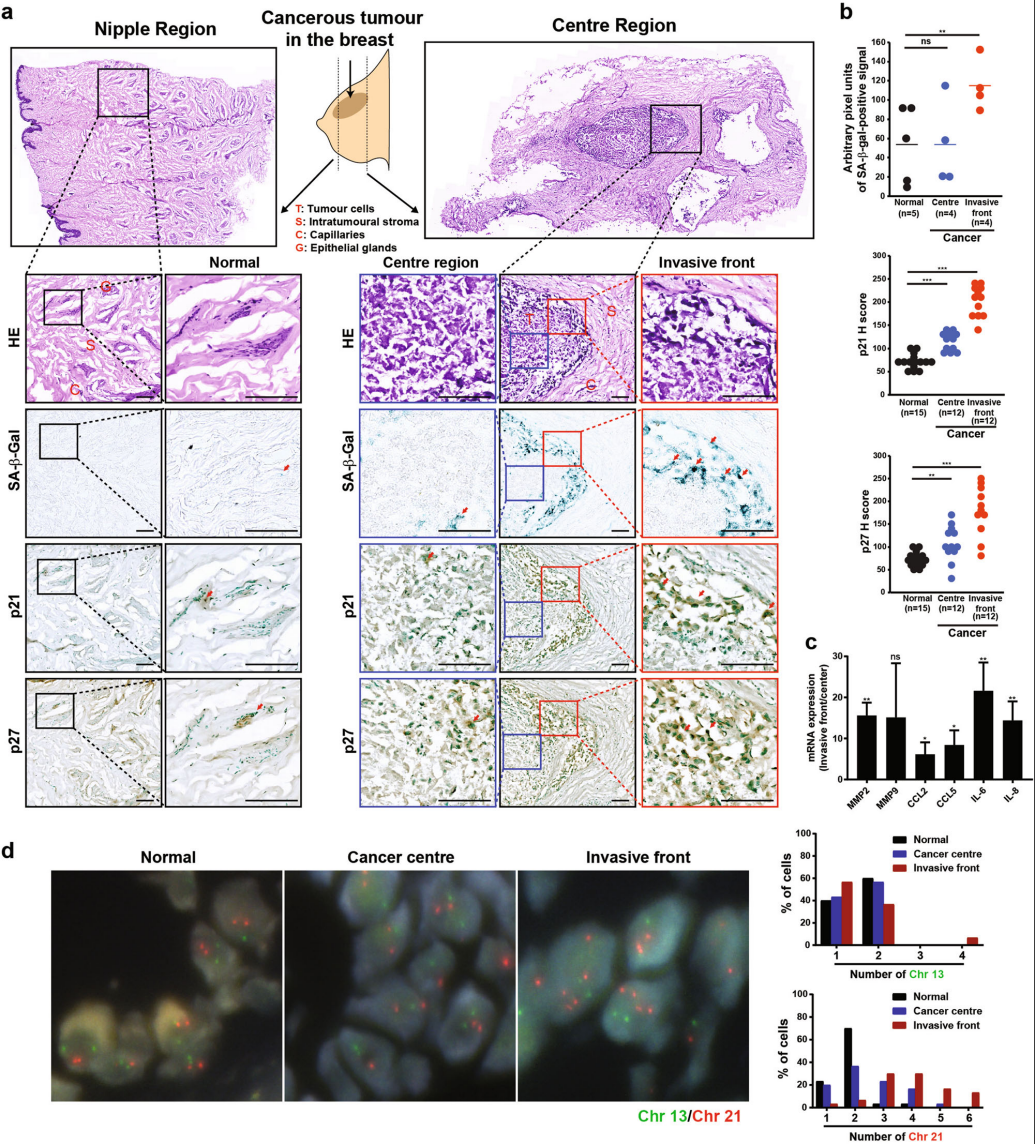
Correlation of increased aneuploidy and senescence at the invasive front in human breast tumours. **a** Tissue sections of breast tumours and corresponding normal nipple regions were stained with SA-β-gal, HE, p21 and p27, or counterstained with methyl green. Images depict normal, cancer centre and invasive front regions. (Left) Normal nipple region (Right) Cancer centre area and cancer invasive front. Red arrows indicate positive staining. Scale bar, 500 µm. **b** Plots show percentages of cells positive for SA-β-gal, p21 and p27 in the normal nipple region, cancer centre area and cancer invasive front as in (**a**). The intensity for SA-β-gal was analysed using ImageJ, and expression of p21 and p27 was analysed using H score as described in Supplementary Table S8. *P < 0.05, **P < 0.01, ***P < 0.001, ****P < 0.0001. NS not significant by Student’s *t*-test. **c** Areas demarcating cancer centre and invasive fronts were dissected by a pathologist for qRT-PCR analysis of target mRNA expression of SASP factors. mRNA levels were normalised to actin. Plot shows relative mRNA expression of cancer invasive fronts compared with corresponding centre. Data are presented as mean ± SD. *P < 0.05, **P < 0.01, ***P < 0.001, ****P < 0.0001. NS not significant by Student’s *t*-test. *n* = 3. **d** Interphase FISH analysis using probes for chromosome 13 (green) and 21 (red) in cancer invasive front and the corresponding cancer centre area and normal region of human breast tumour. (Left) Representative images and (Right) plots showing distribution of FISH signals for chromosomes 13 and 21 in the invasive front and the corresponding centre area and normal region of one breast tumour. *n* = 30 cells

To extend our findings to tumours, we determined possible correlation of high aneuploidy and senescence in patient samples of invasive ductal breast carcinomas. We categorised tissue sections into ‘centre’ and ‘nipple’ regions (Schematically represented in Fig. 7a). As depicted, the centre region consisted mostly of tumour cells, whereas the nipple region consisted mostly of peri-tumoural normal area of mainly capillaries, epithelial glands and stroma. To examine senescence, we performed SA-β-gal staining and immunohistochemical staining to detect p21 and p27 expression. As shown in Fig. 7a, b, we observed a higher incidence of senescence in the centre tumour region compared to the peri-tumoural normal region. Interestingly, we observed senescent cells to be frequently located at the invasive tumour front compared with the centre region. A very recent study in papillary thyroid carcinomas (PTCs) supports our findings^42^; notably, senescent tumour cells frequently present at the invasive borders of PTC displayed invasive ability via the SASP. This prompted us to compare SASP mRNA expression between the tumour centre and invasive front where senescent cells were observed to be predominantly located. The regions of interest from the same tumour samples analysed previously were microscopically dissected by a pathologist. qRT-PCR analysis revealed upregulation of various SASP factors at the invasive front compared with the cancer centre (Fig. 7c), suggesting that SASP may promote paracrine tumour cell invasion in breast cancers.

To test whether higher incidence of aneuploidy correlated with senescence, ploidy was assessed using two-chromosomes FISH (chromosome 13: green and chromosome 21: red) in the same tumour samples analysed previously. Figure 7d shows representative images and quantitation of Chr 13 and Chr 21 signals from one tumour sample, with rest of data in Supplementary Figure S8. FISH analyses revealed greater deviation from the diploid chromosome number (two Chr 13 and two Chr 21 signals) in this order: normal < cancer centre < invasive front (Fig. 7d). Notably, we observed a significant increase of near-tetraploid cells at the invasive front compared with the centre and normal regions. This is consistent with a recent paper showing that near-tetraploid cancer cells exhibit enhanced invasiveness^43^. Importantly, our findings reveal a correlation between increased variation in ploidy and greater degree of senescence/SASP at the invasive front, suggesting that paracrine effects of SASP from aneuploidy-induced senescent cells may plausibly promote invasion and other tumourigenic effects in tumours as well.

**Fig. 8:**
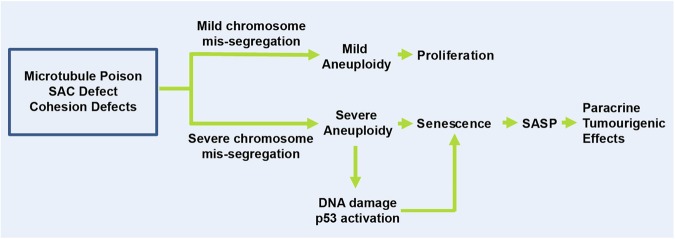
Proposed model of aneuploidy-induced senescence and potential role in tumourigenesis. Different degrees of aneuploidy were induced by engendering chromosome mis-segregation using a microtubule poison, SAC inhibition and cohesion defects. Cells with a low amount of chromosome mis-segregation and mild aneuploidy continue to proliferate. In contrast, cells with severe chromosome mis-segregation and severe aneuploidy enter senescence in a p53-dependent manner. DNA damage and other aneuploidy-induced stresses are associated with senescence induction. Notably, aneuploidy-associated SASP confers paracrine tumourigenic effects such as enhanced angiogenesis and cancer cell migration and invasion

In conclusion, we propose a model where mildly aneuploid cells in response to lower degree of chromosome mis-segregation continue proliferating, whereas severely aneuploid cells are capable of entering a p53-dependent senescence (Fig. 8). Importantly, aneuploidy-induced senescence represents a route by which aneuploidy may promote tumourigenesis, as revealed by the paracrine tumourigenic effects of SASP produced.

## Discussion

CIN is a hallmark of cancers. The strong association between CIN, aneuploidy and cancer conveys the significant role of abnormal ploidy status in contributing to tumour development and drug resistance^3,44–46^. Aneuploidy has recently been associated with poor response to immunotherapy in patients with metastatic melanoma, suggesting that tumour aneuploidy may be a useful prognostication biomarker^47^. Despite the strong correlation of highly aneuploid tumours with poor prognosis and resistance to therapy, there is still much unknown about mechanisms by which CIN and aneuploidy contribute to cancer initiation and progression.

Here we created cell populations with distinguishable random aneuploidies (mild and severe aneuploidies) using chemical and genetic interventions to determine cellular fates post-aneuploidy generation. Our findings indicate that while mildly aneuploid cells ( < 5 chromosomes lost or gained) tend to continue proliferating, severely aneuploid cells (more than five chromosomes lost or gained and polyploid cells) entered senescence. Our results reveal that aneuploidy-induced senescence poses functional consequences for tumour development. Although this may seem like a paradox, as senescence represents a tumour-suppressive mechanism, tumour development can be triggered via the SASP^38,48,49^. The SASP consists of a myriad of soluble factors such as the pro-inflammatory cytokines, chemokines, growth factors and matrix metalloproteinases. These are secreted into the tumour microenvironment and can affect neighbouring cells. Depending on the cell type and context, the SASP can be tumour suppressive^33–36^ or pro-tumourigenic^37–41^. Aneuploidy-induced senescent cells were found to elicit pro-tumourigenic SASP that induced migration, invasion and angiogenesis in neighbouring cells via paracrine action (Fig. 6, Supplementary Figures S6-S7).

Aneuploidy and senescence were found to be present to a greater extent in the tumour region compared with matched normal region in invasive ductal breast carcinomas (Fig. 7). Interestingly, increased aneuploidy and senescence/SASP were observed at the tumour invasive front compared to the centre. Our results are supported by recent evidence in PTCs, demonstrating frequent location of senescent tumour cells at the collective invasive border, as well as higher invasive ability displayed by senescent cells than non-senescent tumour cells via SASP function^42^. Intriguingly, besides conferring paracrine abilities, senescent cells themselves could lead collective invasion and increased cell survival in PTC. Similar to PTC, breast cancer cells also invade with features of collective invasion^50^. Hence, it will be of interest to determine whether aneuploidy-induced senescent cells can also lead collective invasion in breast tumours. It will also be critical to evaluate the extent to which aneuploidy can influence senescence at the tumour invasive front. Overall, our data add a new dimension as to how aneuploidy could potentially promote tumour development.

It is noteworthy that the severely aneuploid cells generated in our study include a significant population of tetraploid and even octoploid cells (Fig. 1c, e, f). During tumourigenesis, tetraploids are often found in pre-cancerous lesions and early stages of some cancers^51,52^. These cells either undergo apoptosis or revert to aneuploid cells with tumourigenic potential^53–55^. In addition, p53-null tetraploid mouse mammary epithelial cells MMEC led to an increase in chromosomal abnormalities, unlike their diploid counterparts^56^. Indeed, polyploid tumour cells have been proposed as critical drug targets for therapy^57,58^. Importantly, a very recent report showed that near-tetraploid cancer cells exhibit enhanced invasiveness^43^, lending further support to our findings. Our study thus describes an additional route by which polyploidization per se could contribute to tumourigenesis via senescence and SASP.

Our data are consistent with recent reports linking chromosome instability to senescence and SASP^59,60^. Studying this in the ageing context, Montagna and colleagues^59^ demonstrated that CIN led to senescence and SASP in human primary fibroblasts. They postulate that this could contribute to age-related pathologies as the CIN-associated secretome could potentially cause bystander normal diploid cells to become senescent via the paracrine pro-senescence effects of the SASP. In addition, in a quest to investigate pathways that limit aneuploid cells from becoming tumourigenic, work from the Amon lab showed that cells with complex karyotypes could undergo senescence^60^. They further showed that such cells produced pro-inflammatory cytokines (SASP components) that promoted their own clearance by natural killer cells. Although aneuploidy-induced senescent cells may be eliminated during tumour development, our study demonstrates that the secretome (SASP) from cells harbouring a higher degree of aneuploidy can confer pro-tumourigenic effects in a paracrine-dependent manner. In this way, one could speculate that even if aneuploidy-induced senescent cells were to be cleared, the SASP once produced, may engender tumourigenic properties on neighbouring cells. Indeed, a recent large-scale analysis study of tumour versus normal samples representing twelve cancer types from The Cancer Genome Atlas (TCGA) project from the Elledge group reported that tumour aneuploidy correlated with immune evasion^47^. The study showed that aneuploidy restricted cytotoxic immune processes during tumourigenesis, and that most tumours with extensive aneuploidy had a reduced number of infiltrating immune cells. How does one reconcile the findings from the Amon lab (immune recognition) and the Elledge lab (immune evasion)? One could speculate that the immunogenic tumour microenvironment may be altered to an immune evasive one at some point during tumour development, or that paracrine effects of aneuploidy-induced SASP emerge predominantly within an immunosuppressive microenvironment. Interestingly, an increase in CIN and aneuploidy has been implicated in age-related pathologies^61,62^. Decreased immune surveillance has been observed with age, which can increase the risk of tumour development^63^. Thus, aneuploidy-induced senescence may also represent a mechanism by which cancer incidence increases with age. An exciting area of study would be to elucidate links between cancer and aging via aneuploidy-induced SASP function from the aged tumour microenvironment perspective.

In light of our findings that a greater degree of aneuploidy, senescence and SASP factors were present at the invasion front of ductal breast tumours, it will be of particular interest to identify pathways influencing the development and function of aneuploidy-induced SASP proteins in controlling paracrine pro-tumourigenic effects. Modulating such pathways may be beneficial in targeting the cell non-autonomous function by which aneuploid cells can contribute to cancers. Further studies from larger patient cohorts would also be required to fully assess the extent to which aneuploidy can influence senescence at the tumour invasive front. Co-expression of genes defective in processes implicated in chromosome mis-segregation (such as the SAC, sister chromatid cohesion and kinetochore assembly) and senescence may serve as useful tools to estimate CIN and malignant potential. Another area of interest relevant for cancer therapy would be to investigate whether aneuploidy-induced senescent cells yield ‘revertants’, an outcome that has been reported to lead to aggressive variants^64^. Studying this could yield further insights into the molecular processes by which aneuploidy-induced senescence contribute to tumour progression, invasion and metastasis and potentially facilitate development of therapies for high-grade tumours.

## Materials and methods

### Cell culture and reagents

Cell lines were purchased from American Type Culture Collection. Cells were maintained in Dulbecco’s modified Eagle’s medium (DMEM)/F-12 (Gibco) (RPE-1), McCoy’s 5A (Gibco) (HCT116) or DMEM (Gibco) (HEK293T and U2OS) supplemented with 10% fetal bovine serum (FBS) (Hyclone, GE Healthcare Life Sciences) and 1% penicillin/streptomycin (Invitrogen). Cells were cultured at 37 °C with 5% CO_2_ in a humidified environment. For cell synchronisation, cells were treated with serum stravation for 24 h, followed by treatment with 3 mM thymidine (Sigma) for 24 and 3 h of drug-free media before drug treatment. Noc (N3000, US Biological) and Rev (Cayman Chemicals) were used to induce aneuploidy.

### shRNA lentiviral transduction

Lentivirus was produced from HEK293T cells by transfecting pLKO.1 (control shRNA), pLKO.1-shp53, pLKO.1-shBUB1 or pLKO.1-shSMC1A together with lentiviral packaging plasmids. RPE-1 cells were infected with lentivirus for 48 h with 8 µg/ml of polybrene. Cells were then selected with 5 µg/ml puromycin for 10 days and used for further analysis. Information of shRNA plasmids can be found in Supplementary Table S4.

### RNA-seq and analyses

RNA-seq reads were aligned to the human genome by Tophat 2.1.0^65^. Quality control of single-ended reads was performed by RNA-SeQC (1.1.7)^66^. The quality control results were all within acceptable ranges. Cuffdiff (2.2.1)^67^ was used to quantify expression and detect differentially expressed genes with gene annotation GTF file downloaded from Ensembl (version GRCh37.75)^68^. For each comparison between two conditions, genes with fold change at least two folds and *q*-value < 0.05 were selected as significantly differentially expressed genes. PCA was performed on two biological replicates using function PCAplot from R package CummeRbund (v2.8.2). GO analysis for differentially expressed genes was conducted using online database DAVID (Version 6.7) ^26^.

### Quantitative real-time PCR

Total RNA was extracted using RNeasy plus Mini Kit (Qiagen). RNA was reverse-transcribed using iScript^TM^ RT supermix (Bio-Rad). qRT-PCR was performed using SYBR® Select Master Mix for CFX (Life Technologies, #4472942) by StepOnePlus™ Real-Time PCR System (Applied Biosystem). Primers used are in Supplementary Tables S5-S6.

### Live-cell imaging for visualisation of lagging chromosomes

See Supplementary Methods for detailed procedure.

### Flow cytometry

See Supplementary Methods for detailed procedure.

### IF microscopy

See Supplementary Methods for detailed procedure.

### Growth proliferation assay

See Supplementary Methods for detailed procedure.

### Cell viability assay

See Supplementary Methods for detailed procedure.

### Western blotting

Cells were lysed in RIPA buffer (Thermo Scientific) with protease and phosphatase inhibitor cocktail (Thermo Scientific). After electrophoresis and transfer, membranes were incubated with primary antibodies overnight at 4 °C followed by horseradish peroxidase-conjugated secondary antibodies (1:10 000, GE Healthcare). Antibodies used are listed in Supplementary Table S7.

### Chromosome spreads

Chromosome spreads were performed as described previously [19].

### SA-β-gal staining

SA-β-gal staining was performed using the Senescence β-galactosidase Staining Kit (Cell Signaling Technology #9860) following the manufacturer’s instructions.

### BrdU incorporation assay

Cells seeded on glass coverslips were incubated with BrdU Labeling and Detection Kit I (Roche #11296736001) for 24 h and visualised according to the manufacturer’s instructions.

### Fluorescence in situ hybridisation

Cells were trypsinized and collected by centrifugation, then fixed with ice-cold Carnoy’s fixative and dropped onto slides. FISH analysis was performed following the manufacturer’s instructions using either two centromere-specific probes for chromosome 6 (green) and 8 (red) (Cytocell Aquarius #LPE006G and LPE008R) or FISH probe set with locus-specific XA13 (green)/18 (aqua)/21(red) (MetaSystems Probes #D-5607-100-TC). Slides were then counterstained with 4′,6-diamidino-2-phenylindole for imaging with either a Nikon Eclipse-Ti microscope or the automated slide scanner system (MetaSystems) using classifier Metacyte spotcount XA131821. FISH experiments on 2 µm-thick cryosections from human breast tissues were performed using the same FISH probe set XA13(green)/18(aqua)/21(red) by Advanced Molecular Pathology Laboratory at SingHealth; only chromosomes 13 and 21 were analysed.

### CM preparation

To generate CM from control and 16 h Noc-induced aneuploid RPE-1 cells, cells were incubated with media supplemented with 0.5% FBS and 1% penicillin/streptomycin. Five days later, CM was collected and filtered with 0.22 µm pore filters. To generate CM from control and 1 µM Rev-treated RPE-1 and HCT116 cells, cells were grown in fresh media for two more days after drug washout, then replated into media supplemented with 0.5% FBS. Three days later, CM was collected. For wound-healing assay, CM containing 0.5% FBS was used. For transwell invasion assay and cellular migration and invasion in zebrafish assay, CM containing 0.5% FBS was mixed with media containing 40% FBS in a proportion of 3:1 to prepare CM containing 10% FBS.

### Wound-healing assay

U2OS cells seeded in six-well plates were incubated with indicated CM for 24 h. Confluent monolayer was scratched with a 200 µl pipette tip to generate a wound. Live-cell imaging was performed with × 10 objective lens. Phase-contrast images were taken at multiple points every 1 h for 24–48 h and analysed using ImageJ.

### Transwell invasion assay

U2OS cells were incubated with CM for 2 days, trypsinized, resuspended in CM and plated onto transwell inserts (8 µm pore size) pre-coated or uncoated with Matrigel (BD Biosciences) with 20% FBS added to the lower chamber. Twenty-four hours later, non-invasive cells on the upper surface of the inserts were removed. Invasive cells on the lower surface were fixed, stained with crystal violet and observed under × 10 objective. The number of cells in six to nine random fields were counted per condition. The percentage of invasive cells was determined as follows: invasive cells (%) = mean number of cells invading through Matrigel matrix insert membrane/mean number of cells migrating through uncoated insert membrane × 100%.

### Multiplex cytokine analysis

Forty-one analytes from Human Cytokine Panel 1 (Merck Millipore) were measured according to the manufacturer’s instructions. Plates were washed using Tecan Hydrospeed Washer (Tecan) and read with Flexmap 3D system (Luminex Corp). Data were analysed using Bio-Plex manager 6.0 (Bio-Rad) with a five-parameter curve-fitting algorithm applied for standard curve calculations.

### Choroid sprouting assay

The choroid tissue from P3 mice eyes was separated in 10% FBS–phosphate-buffered saline solution. The peripheral choroid layer was isolated from the retina and cut into 1 mm × 1 mm segments. Each segment was mounted on a 20 µl drop of Matrigel and incubated with respective CM containing 0.5% FBS in 1:3 diluted with EGM2 media (Lonza) for 4 days. Media were changed every other day. On Day 4, phase-contrast images of individual explants were taken using a Nikon Eclipse-Ti microscope. The ratio of area of vascular sprouting vs area of the explant was analysed using ImageJ.

### Cellular migration and invasion in zebrafish (ZgraftTM)

U2OS or HCT116 cells were treated with CM for 2 days. Cells were stained with red-fluorescent dye DiI (Vybrant, Life Technologies) and subsequently injected into 48 h post-fertilisation zebrafish embryos. Approximately 2 h after injection, embryos with fluorescent cells outside of the yolk sac were excluded from further experimentation and analysis. Four days later, embryos were imaged to determine metastatic tumour foci position relative to injection site. Data were blindly obtained and analysed. Detailed procedure is provided in Supplementary Methods.

### Patient breast cancer tissues

Biopsies were collected using mastectomy procedure and were snap-frozen in liquid nitrogen immediately after surgery. Frozen biopsies were thawed slowly, fixed and processed accordingly for analytical study. All human breast tissue samples were collected prospectively from patients undergoing surgery at the National Cancer Centre, Singapore. None of the patients underwent prior chemotherapy. The selected samples were de-identified, stored and analysed according to procedures described in protocols #CIRB 2015/2976 and #IRB-2018-04-009, approved by SingHealth CIRB and NTU IRB. Informed consents were obtained from all patients.

### Immunohistochemistry

For cryosectioned tissue, 4 µm-thick sections were fixed and incubated with primary antibodies overnight at 4°C, followed by incubation with biotinylated secondary antibodies (Vector Laboratories, USA). Sections were then incubated in Avidin:Biotinylated enzyme Complex (Vector Laboratories) for 30 min, developed with 3,3’-diaminobenzidine substrate (Vector Laboratories) and nuclei counterstained with methyl green. Slides were mounted with Fluka Eukitt® quick-hardening mounting medium (Sigma-Aldrich, USA). See Supplementary Methods for detailed procedure.

### Statistical analysis

All experiments were repeated at least three times unless otherwise stated. Data were blindly obtained and analysed. Data are expressed as mean ± SD or mean ± SEM as indicated. GraphPad Prism 6 was used to analyse all the data. Student’s *t*-test (two-tailed) was used to determine the statistical differences between different groups (*P < 0.05, **P < 0.01, ***P < 0.001, ****P < 0.0001, and NS not significant).

### Data availability

RNA-seq data have been deposited in the NCBI Gene Expression Omnibus repository (http://www.ncbi.nlm.nih.gov/geo/) under the accession number GSE109519. Private link with the key ‘ctulcymuhxorjgp’ for the reviewer until acceptance of the manuscript.

## Electronic supplementary material

Supplementary Methods

Supplementary Figure Legends

Supplementary Figure S1

Supplementary Figure S2

Supplementary Figure S3

Supplementary Figure S4

Supplementary Figure S5

Supplementary Figure S6

Supplementary Figure S7

Supplementary Figure S8

Supplementary Table S1

Supplementary Table S2

Supplementary Table S3

Supplementary Table S4-S8

## References

[1] Weaver BA, Cleveland DW (2006). Does aneuploidy cause cancer?. Curr. Opin. Cell Biol..

[2] Risques RA (2001). Redefining the significance of aneuploidy in the prognostic assessment of colorectal cancer. Lab. Invest..

[3] Zasadil LM, Britigan EM, Weaver BA (2013). 2n or not 2n: Aneuploidy, polyploidy and chromosomal instability in primary and tumor cells. Semin. Cell. Dev. Biol..

[4] Carter SL, Eklund AC, Kohane IS, Harris LN, Szallasi Z (2006). A signature of chromosomal instability inferred from gene expression profiles predicts clinical outcome in multiple human cancers. Nat. Genet..

[5] Lagarde P (2012). Mitotic checkpoints and chromosome instability are strong predictors of clinical outcome in gastrointestinal stromal tumors. Clin. Cancer Res..

[6] Thompson SL, Compton DA (2008). Examining the link between chromosomal instability and aneuploidy in human cells. J. Cell Biol..

[7] Holland AJ, Cleveland DW (2009). Boveri revisited: chromosomal instability, aneuploidy and tumorigenesis. Nat. Rev. Mol. Cell Biol..

[8] Thompson SL, Bakhoum SF, Compton DA (2010). Mechanisms of chromosomal instability. Curr. Biol..

[9] Weaver BA, Silk AD, Montagna C, Verdier-Pinard P, Cleveland DW (2007). Aneuploidy acts both oncogenically and as a tumor suppressor. Cancer Cell..

[10] Sotillo R (2007). Mad2 overexpression promotes aneuploidy and tumorigenesis in mice. Cancer Cell..

[11] Hanks S (2004). Constitutional aneuploidy and cancer predisposition caused by biallelic mutations in BUB1B. Nat. Genet..

[12] Snape K (2011). Mutations in CEP57 cause mosaic variegated aneuploidy syndrome. Nat. Genet..

[13] Orr B, Talje L, Liu Z, Kwok BH, Compton DA (2016). Adaptive resistance to an inhibitor of chromosomal instability in human cancer cells. Cell Rep..

[14] Laughney AM, Elizalde S, Genovese G, Bakhoum SF (2015). Dynamics of tumor heterogeneity derived from clonal karyotypic evolution. Cell Rep..

[15] Li R, Hehlman R, Sachs R, Duesberg P (2005). Chromosomal alterations cause the high rates and wide ranges of drug resistance in cancer cells. Cancer Genet. Cytogenet..

[16] Baker DJ, Jin F, Jeganathan KB, van Deursen JM (2009). Whole chromosome instability caused by Bub1 insufficiency drives tumorigenesis through tumor suppressor gene loss of heterozygosity. Cancer Cell..

[17] Passerini V (2016). The presence of extra chromosomes leads to genomic instability. Nat. Commun..

[18] Janssen A, van der Burg M, Szuhai K, Kops GJ, Medema RH (2011). Chromosome segregation errors as a cause of DNA damage and structural chromosome aberrations. Science.

[19] Crasta K (2012). DNA breaks and chromosome pulverization from errors in mitosis. Nature.

[20] Sheltzer JM, Torres EM, Dunham MJ, Amon A (2012). Transcriptional consequences of aneuploidy. Proc. Natl Acad. Sci. USA.

[21] Durrbaum M (2014). Unique features of the transcriptional response to model aneuploidy in human cells. BMC Genomics.

[22] Cimini D (2001). Merotelic kinetochore orientation is a major mechanism of aneuploidy in mitotic mammalian tissue cells. J. Cell Biol..

[23] Santaguida S, Tighe A, D’Alise AM, Taylor SS, Musacchio A (2010). Dissecting the role of MPS1 in chromosome biorientation and the spindle checkpoint through the small molecule inhibitor reversine. J. Cell Biol..

[24] Thompson SL, Compton DA (2010). Proliferation of aneuploid human cells is limited by a p53-dependent mechanism. J. Cell Biol..

[25] Santaguida S, Vasile E, White E, Amon A (2015). Aneuploidy-induced cellular stresses limit autophagic degradation. Genes Dev..

[26] Huang da W, Sherman BT, Lempicki RA (2009). Systematic and integrative analysis of large gene lists using DAVID bioinformatics resources. Nat. Protoc..

[27] Fulda S, Gorman AM, Hori O, Samali A (2010). Cellular stress responses: cell survival and cell death. Int. J. Cell Biol..

[28] Kuo LJ, Yang LX (2008). Gamma-H2AX - a novel biomarker for DNA double-strand breaks. In Vivo.

[29] Johmura Y, Nakanishi M (2016). Multiple facets of p53 in senescence induction and maintenance. Cancer Sci..

[30] te Poele RH, Okorokov AL, Jardine L, Cummings J, Joel SP (2002). DNA damage is able to induce senescence in tumor cells in vitro and in vivo. Cancer Res..

[31] Serrano M, Lin AW, McCurrach ME, Beach D, Lowe SW (1997). Oncogenic ras provokes premature cell senescence associated with accumulation of p53 and p16INK4a. Cell.

[32] Pawlikowski JS, Adams PD, Nelson DM (2013). Senescence at a glance. J. Cell Sci..

[33] Acosta JC (2008). Chemokine signaling via the CXCR2 receptor reinforces senescence. Cell.

[34] Kuilman T (2008). Oncogene-induced senescence relayed by an interleukin-dependent inflammatory network. Cell.

[35] Xue W (2007). Senescence and tumour clearance is triggered by p53 restoration in murine liver carcinomas. Nature.

[36] Kang TW (2011). Senescence surveillance of pre-malignant hepatocytes limits liver cancer development. Nature.

[37] Coppe JP (2008). Senescence-associated secretory phenotypes reveal cell-nonautonomous functions of oncogenic RAS and the p53 tumor suppressor. PLoS Biol..

[38] Krtolica A, Parrinello S, Lockett S, Desprez PY, Campisi J (2001). Senescent fibroblasts promote epithelial cell growth and tumorigenesis: a link between cancer and aging. Proc. Natl Acad. Sci. USA.

[39] Liu D, Hornsby PJ (2007). Senescent human fibroblasts increase the early growth of xenograft tumors via matrix metalloproteinase secretion. Cancer Res..

[40] Bavik C (2006). The gene expression program of prostate fibroblast senescence modulates neoplastic epithelial cell proliferation through paracrine mechanisms. Cancer Res..

[41] Davalos AR, Coppe JP, Campisi J, Desprez PY (2010). Senescent cells as a source of inflammatory factors for tumor progression. Cancer Metastas-. Rev..

[42] Kim YH (2017). Senescent tumor cells lead the collective invasion in thyroid cancer. Nat. Commun..

[43] Wangsa D., et al. Near-tetraploid cancer cells show chromosome instability triggered by replication stress and exhibit enhanced invasiveness. *FASEB J*. 32, 3502–3517 (2018). 10.1096/fj.201700247RR PMC5998968 29452566

[44] McGranahan N, Burrell RA, Endesfelder D, Novelli MR, Swanton C (2012). Cancer chromosomal instability: therapeutic and diagnostic challenges. EMBO Rep..

[45] Holland AJ, Cleveland DW (2012). Losing balance: the origin and impact of aneuploidy in cancer. EMBO Rep..

[46] Giam M, Rancati G (2015). Aneuploidy and chromosomal instability in cancer: a jackpot to chaos. Cell. Div..

[47] Davoli T, Uno H, Wooten EC, Elledge SJ (2017). Tumor aneuploidy correlates with markers of immune evasion and with reduced response to immunotherapy. Science.

[48] Canino C (2012). SASP mediates chemoresistance and tumor-initiating-activity of mesothelioma cells. Oncogene.

[49] Yoshimoto S (2013). Obesity-induced gut microbial metabolite promotes liver cancer through senescence secretome. Nature.

[50] Cheung KJ, Gabrielson E, Werb Z, Ewald AJ (2013). Collective invasion in breast cancer requires a conserved basal epithelial program. Cell.

[51] Margolis RL (2005). Tetraploidy and tumor development. Cancer Cell..

[52] Shackney SE (1989). Model for the genetic evolution of human solid tumors. Cancer Res..

[53] Dewhurst SM (2014). Tolerance of whole-genome doubling propagates chromosomal instability and accelerates cancer genome evolution. Cancer Discov..

[54] Davoli T, de Lange T (2012). Telomere-driven tetraploidization occurs in human cells undergoing crisis and promotes transformation of mouse cells. Cancer Cell..

[55] Margolis RL, Lohez OD, Andreassen PR (2003). G1 tetraploidy checkpoint and the suppression of tumorigenesis. J. Cell. Biochem..

[56] Fujiwara T (2005). Cytokinesis failure generating tetraploids promotes tumorigenesis in p53-null cells. Nature.

[57] Coward J, Harding A (2014). Size does matter: why polyploid tumor cells are critical drug targets in the war on cancer. Front. Oncol..

[58] Vitale I (2011). Illicit survival of cancer cells during polyploidization and depolyploidization. Cell Death Differ..

[59] Andriani GA (2016). Whole chromosome instability induces senescence and promotes SASP. Sci. Rep..

[60] Santaguida S (2017). Chromosome mis-segregation generates cell-cycle-arrested cells with complex karyotypes that are eliminated by the immune system. Dev. Cell..

[61] Baker DJ (2013). Increased expression of BubR1 protects against aneuploidy and cancer and extends healthy lifespan. Nat. Cell Biol..

[62] Ricke RM, van Deursen JM (2013). Aneuploidy in health, disease, and aging. J. Cell Biol..

[63] Derhovanessian E, Solana R, Larbi A, Pawelec G (2008). Immunity, ageing and cancer. Immun. Ageing..

[64] Yang L, Fang J, Chen J (2017). Tumor cell senescence response produces aggressive variants. Cell Death Discov..

[65] Trapnell C, Pachter L, Salzberg SL (2009). TopHat: discovering splice junctions with RNA-Seq. Bioinformatics.

[66] DeLuca DS (2012). RNA-SeQC: RNA-seq metrics for quality control and process optimization. Bioinformatics.

[67] Trapnell C (2012). Differential gene and transcript expression analysis of RNA-seq experiments with TopHat and Cufflinks. Nat. Protoc..

[68] Hubbard TJ (2009). Ensembl 2009. Nucleic Acids Res.

