# Supplementary material for: Chromosomal instability-induced senescence potentiates cell non-autonomous tumourigenic effects
Source: Oncogenesis. 2018 Aug 15;7(8):62. doi: 10.1038/s41389-018-0072-4 (PMC6092349; doi:10.1038/s41389-018-0072-4)
Supplement: Supplementary file 1 — Supplementary Methods [file 41389_2018_72_MOESM1_ESM.docx]

**SUPPLEMENTARY METHODS**

**Live-cell imaging for visualization of lagging chromosomes**

RPE-1 H2B-GFP cells were grown on glass-bottom 6-well plate (MatTek) and imaged on a Nikon inverted fluorescence microscope equipped with EMCCD camera (Photometrics Evolve 512), a precision motorized stage and Nikon Perfect Focus. Microscope was enclosed within temperature- and CO_2_-controlled environment which maintained an atmosphere of 37°C and 5% humidified CO_2_. For nocodazole (Noc) treatment, after treatment with Noc for 8 h and 16 h respectively, cells were washed with fresh media twice and replaced with fresh media, then imaged immediately under the microscope with the 60× objective. GFP images were captured at multiple points every 3.5 min for 1.5 h - 3 h. Control cells were imaged 9 h after release from thymidine. For reversine (Rev) treatment, synchronised cells were treated with DMSO, 0.2 µM or 1 µM Rev and filmed immediately with a 40× objective. Images were acquired every 5 min for 24 h. Images captured from each experiment were analysed using MetaMorph software (Molecular Devices).

**Flow cytometry**

Cells were fixed with ice-cold 70% ethanol and stored at -20°C. For phospho-Histone H3 staining, fixed cells were washed once with PBS containing 0.5% BSA and then incubated with phospho-Histone H3 (Ser10) antibody conjugated with Alexa Fluor 488 (Cell Signaling Technology #3465S) diluted in PBS containing 0.5% BSA (1:500) for 1 h in the dark at room temperature. Cells were then washed with PBS containing 0.5% BSA and incubated with 250 μg/ml RNase A and 10 μg/ml propidium iodide (PI) at 37°C for 30 min. Analyses were performed using a BD Acurri C6 cytometer (BD Biosciences) and data analysed using FlowJo^®^ (FlowJo, LLC).

**Immunofluorescence microscopy**

Cells plated on glass coverslips in 6-well plates were fixed with 4% paraformaldehyde for 15 min. Cells were then blocked with blocking buffer (PBS containing 2% BSA, 5% FBS and 0.1% Triton X-100) for 30 min and incubated with anti-phospho-Histone H2AX (Ser139) (Merck Millipore #05-636, 1:500) overnight at 4°C. Cells were washed thrice with PBS containing 0.1% Triton X-100 and incubated with fluorescence-conjugated secondary antibody (Invitrogen, 1:1000) for 1 h. Coverslips were then counterstained with Hoechst 33342 (Invitrogen), mounted onto slides with FluorSave^TM^ Reagent (Calbiochem), and imaged using a iLAS2 Pulse FRAP/Ablation System microscope with a 60× objective.

**Growth proliferation assay**

Cells were plated in 6-well plates at 5×10^4^ cells/well (U2OS) or 1.0×10^5^ cells/well (HCT116) and grown for 24 h. Cells were then washed twice with PBS and incubated with indicated CM containing 10% FBS for up to four days. For Day 4 samples, media were replenished once during the experiment. Cell number on each day was then counted.

**Cell viability assay**

U2OS cells were plated in 96-well plate and incubated with indicated CM for 72 h. Cell viability was assessed using the CellTiter 96^®^ AQueous One Solution Cell Proliferation Assay (Promega #G3580) according to the manufacturer's instructions.

**Cellular migration and invasion in zebrafish** **(ZgraftTM)**

The Zebrafish Research Facility as well as the Animal Care Committee in Acenzia Inc. is certified through Ontario Ministry of Agriculture, Food and Rural Affairs (OMAFRA) and all protocols used in the laboratories were monitored by the Animal Health and Welfare Branch, OMAFRA. U2OS or HCT116 cells were treated with CM for two days. Cells were incubated in serum-free media containing red-fluorescent dye DiI (Vybrant, Life Technologies) for 45 min at 37°C, and subsequently injected into 48 h post-fertilization zebrafish embryos. 100 - 200 cells were injected into the yolk sac of embryos at 35°C. Approximately 2 h after injection, embryos with fluorescent cells outside of the yolk sac were excluded from further experimentation and analysis. The rest were incubated for four days post-injection. Each larva was anesthetized by immersion in system water containing tricaine and placed on a 1% agarose bed under an inverted fluorescence microscope for imaging (Leica M165 FC). Images were gathered, aligned to a specific orientation and analysed using ImageJ software to determine metastatic tumour foci position relative to injection site (0,0 on graph). Data were blindly obtained and analysed.

**Immunohistochemistry**

Breast cancer tissue of 4 µm-thickness was sectioned. For cryosectioned tissue, sections were fixed in 4% paraformaldehyde and washed in PBS. Tissue endogenous peroxidase activity was blocked using 3% hydrogen peroxide for 15 min at room temperature. Blocking was performed using 10% normal goat serum (NGS), 1% bovine serum albumin (BSA) containing 0.1% Triton X-100 for 1 h at room temperature. Sections were then incubated with primary antibodies as follows: anti human p21 Waf1/Cip1 (12D1) (1:100, Cell Signaling Technology #2947S); anti human p27 KIP1 (1:200, Abcam ab193379) in PBS containing 3% NGS overnight at 4 ºC, followed by incubation with biotinylated secondary antibodies (Vector laboratories, USA). Sections were then incubated in Avidin:Biotinylated enzyme Complex (ABC; Vector Laboratories, USA) for 30 min, developed with 3,3’-diaminobenzidine (DAB) substrate (Vector Laboratories, USA) to give a brown colour stain and nuclei counterstained with methyl green. Slides were mounted with Fluka Eukitt® quick-hardening mounting medium (Sigma-Aldrich, USA). Images were taken using Carl Zeiss slide scanner Axio Scan.Z1 using a Plan-Apochromat 40x/0.95 NA objective and Hitachi HV F202, and ZEN 2 slidescan software.
