# Supplementary material for: Chromosomal instability-induced senescence potentiates cell non-autonomous tumourigenic effects
Source: Oncogenesis. 2018 Aug 15;7(8):62. doi: 10.1038/s41389-018-0072-4 (PMC6092349; doi:10.1038/s41389-018-0072-4)
Supplement: Supplementary file 2 — Supplementary Figure Legends [file 41389_2018_72_MOESM2_ESM.docx]

**SUPPLEMENTARY FIGURE LEGENDS**

**Supplementary Figure S1. Nocodazole causes chromosome mis-segregation, aneuploidy and cell cycle arrest.** (**a**) Scheme for time-lapse microscopy after nocodazole (Noc) treatment in RPE-1 H2B-GFP cells. (**b**) (Left) Representative anaphase images depicting lagging chromosomes in RPE-1 H2B-GFP cells treated as per (a). Yellow arrows indicate lagging chromosomes. (Right) Plot shows the percentage of abnormal mitosis after indicated treatments. Abnormal mitosis includes lagging chromosomes, misaligned chromosomes, broken chromatids, chromosome bridges and cytokinetic failure events. At least 20 cells were analysed per condition. Scale bar, 5 µm. (**c**) DAPI staining shows micronucleated and multinucleated cells one day (D1) after mitotic shake-off from Noc treatment in RPE-1 cells. Yellow and white arrows indicate micronucleated and multinucleated cells, respectively. Plot shows percentage of micronucleated and multinucleated cells. Scale bar, 10 µm. At least 100 cells were quantified per condition. (**d**) RPE-1 cells treated with Noc as per Figure 1a were co-stained with propidium iodide (PI) and FITC-conjugated anti-phosphorylated Histone H3 antibody and subjected to flow cytometry. Numbers in each quarter indicate the percentage of different cell populations. FACS profiles on the right show cells stained with PI to determine DNA content. (**e**) Levels of p53 and p21 were determined by Western blotting in RPE-1 cells treated with Noc for 8 h and 16 h, followed by mitotic shake-off and culture in drug-free media for 24 h. Actin was used as loading control. (**f**) Western blot of shRNA-mediated knockdown of p53 in RPE-1 cells. GAPDH was used as loading control. Data are expressed as mean ± SD of three independent experiments. **P*<0.05, ***P*<0.01, ****P*<0.001 and *****P*<0.0001 by Student's *t*-test.

**Supplementary Figure S2. Reversine causes chromosome mis-segregation, aneuploidy and cell cycle arrest.** (**a**) Scheme for time-lapse microscopy after reversine (Rev) treatment in RPE-1 H2B-GFP cells. (**b**) Representative anaphase images of DMSO-treated and Rev-treated RPE-1 H2B-GFP cells. Plot shows percentage of abnormal mitosis after different treatment. Scale bar, 5 µm. Minimum of 30 cells were analysed per condition. (**c-d**) RPE-1 cells treated with Rev as per Figure 1b were subjected to (c) DAPI staining and (d) flow cytometry. Scale bar, 10 µm. Yellow and white arrows indicate micronucleated and multinucleated cells, respectively. (**e-f**) Levels of p53 and p21 in (e) RPE-1 and (f) HCT116 cells after treatment with DMSO or Rev for 24 h. GAPDH was used as loading control. Data are expressed as mean ± SD of three independent experiments. **P*<0.05, ***P*<0.01, *****P*<0.0001; ns, not significant by Student's *t*-test.

**Supplementary Figure S3**. **Aneuploidy-induced senescence is associated with DNA damage.** (**a**) RPE-1 cells were treated with Noc for 8 h or 16 h, followed by mitotic shake-off and culture in fresh media for 1, 3 or 5 days. Cells were then stained for DNA damage marker γH2AX. (Left) Representative images of cells stained for γH2AX of Day 1 samples. (Right) Plot shows percentage of cells containing one or more γH2AX foci. Scale bar, 10 µm. Data are expressed as mean ± SD of three independent experiments. At least 100 cells were quantified for each condition. *P* values were derived from Student's *t*-test (**P*<0.05, ****P*<0.001; ns, not significant). (**b-e**) Western blot analyses of DNA damage marker γH2AX and senescence-associated markers in (b) Noc or (c) Rev-treated RPE-1 cells, (d) Rev-treated HCT116 cells and (e) RPE-1 cells depleted of Bub1 and SMC1A. GAPDH was used as loading control. Synchronized cells treated with 100 ng/ml doxorubicin for 24 h served as positive control for DNA damage in (b). Dox represents doxorubicin.

**Supplementary Figure S4. Loss of p53 promotes SASP expression.** (**a-b**) qRT-PCR analysis of SASP components in (a) RPE-1 shCtrl and shp53 cells and (b) HCT116 WT and p53-null cells 5 days after Noc 16 h or 1 µM Rev treatment. mRNA levels were normalized to actin. Data are expressed as mean ± SEM of four independent experiments.

**Supplementary Figure S5. Secreted factors from aneuploidy-induced senescent cells enhance cell migration without affecting cell viability and proliferation.** (**a**) Cell number of U2OS cells cultured for indicated duration with CM from (left) control or 16 h Noc-treated or (right) control or 1 µM Rev-treated RPE-1 cells. (**b**) Cell viability assay of U2OS cells cultured with indicated CM for 3 days. All data are expressed as mean ± SD of three independent experiments. ns, not significant by Student's *t*-test.

**Supplementary Figure S6. shSMC1A-induced aneuploidy promotes the expression of SASP factors that enhance cell migration.** (**a**) qRT-PCR analysis of SASP components in RPE-1 shCtrl and shSMC1A-2 cells. mRNA levels were normalized to actin. (**b**) RPE-1 cells were infected with lentivirus containing pLKO.1 (shCtrl) or shSMC1A-2 for 48 h and followed by puromycin selection for 10 days. Cells were then washed with PBS and cultured in media containing 0.5% FBS for three days. Conditioned media (CM) were collected and used for wound healing assay on U2OS cells. U2OS cells were incubated with indicated CM for 24 h and subjected to wound healing assay. (Left) Representative images are shown. Scale bar, 200 µm. (Right) Plot shows the percentage of wound area relative to the initial wound area at time 0 h (immediately after the scratch). Data are expressed as mean ± SD from three independent experiments. ***P*<0.01, ****P*<0.001 and *****P*<0.0001 by Student's *t*-test.

**Supplementary Figure S7. Aneuploidy-induced senescent cells enhance cell migration and invasion *in vivo*.** (**a**) HCT116 cells were treated for 2 days with CM from control or 1 µM Rev-treated HCT116 cells and injected into zebrafish embryos. White arrows indicate DiI-stained U2OS cells that invaded the zebrafish body. Plot shows Invasion index and Migration index. Data are expressed as mean ± SEM from three independent experiments. (**b**) U2OS cells were treated for 2 days with CM from control or 1 µM Rev-treated RPE-1 cells followed by injection into zebrafish embryos. White arrows indicate DiI-stained U2OS cells that invaded the zebrafish body. Plot shows invasion and migration index. Data are expressed as mean ± SEM from three independent experiments. (**c-d**) Cell number of U2OS cells cultured for indicated duration with CM from control or 1 µM Rev-treated (c) HCT116 and (d) RPE-1 cells. Data are represented as mean ± SD of three independent experiments. (**e**) Cell number of HCT116 cells cultured for indicated duration with CM generated from control or 1 µM Rev-treated HCT116 cells. Data are expressed as mean ± SD of three independent experiments. All *P* values were derived from Student's *t*-test: **P*<0.05, ***P*<0.01, ****P*<0.001, *****P*<0.0001; ns, not significant.

**Supplementary Figure S8. Prevalence of aneuploid cells at the invasive front in human breast tumours.** Distribution of probe signals for (top panel) chromosome 13 (green) and (bottom panel) chromosome 21 (red) in three cancer invasive fronts and their corresponding centre and normal regions from tissues obtained from patients with invasive ductal breast carcinomas. n=30 cells per condition.
