# Supplementary figures and images for: Chromosomal instability-induced senescence potentiates cell non-autonomous tumourigenic effects
Source: Oncogenesis. 2018 Aug 15;7(8):62. doi: 10.1038/s41389-018-0072-4 (PMC6092349; doi:10.1038/s41389-018-0072-4)

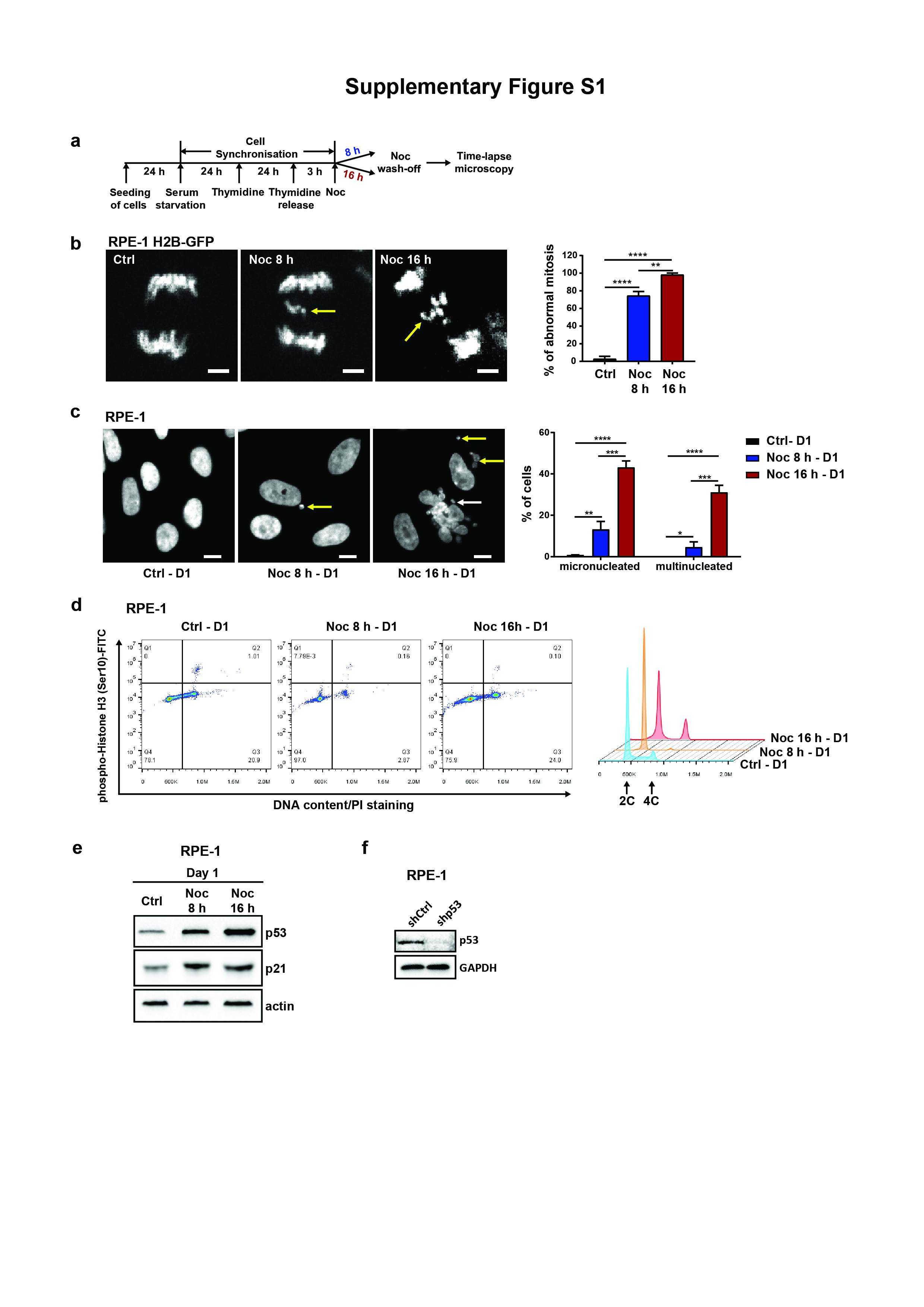

Supplement: Supplementary file 3 — Supplementary Figure S1 [file 41389_2018_72_MOESM3_ESM.jpg]

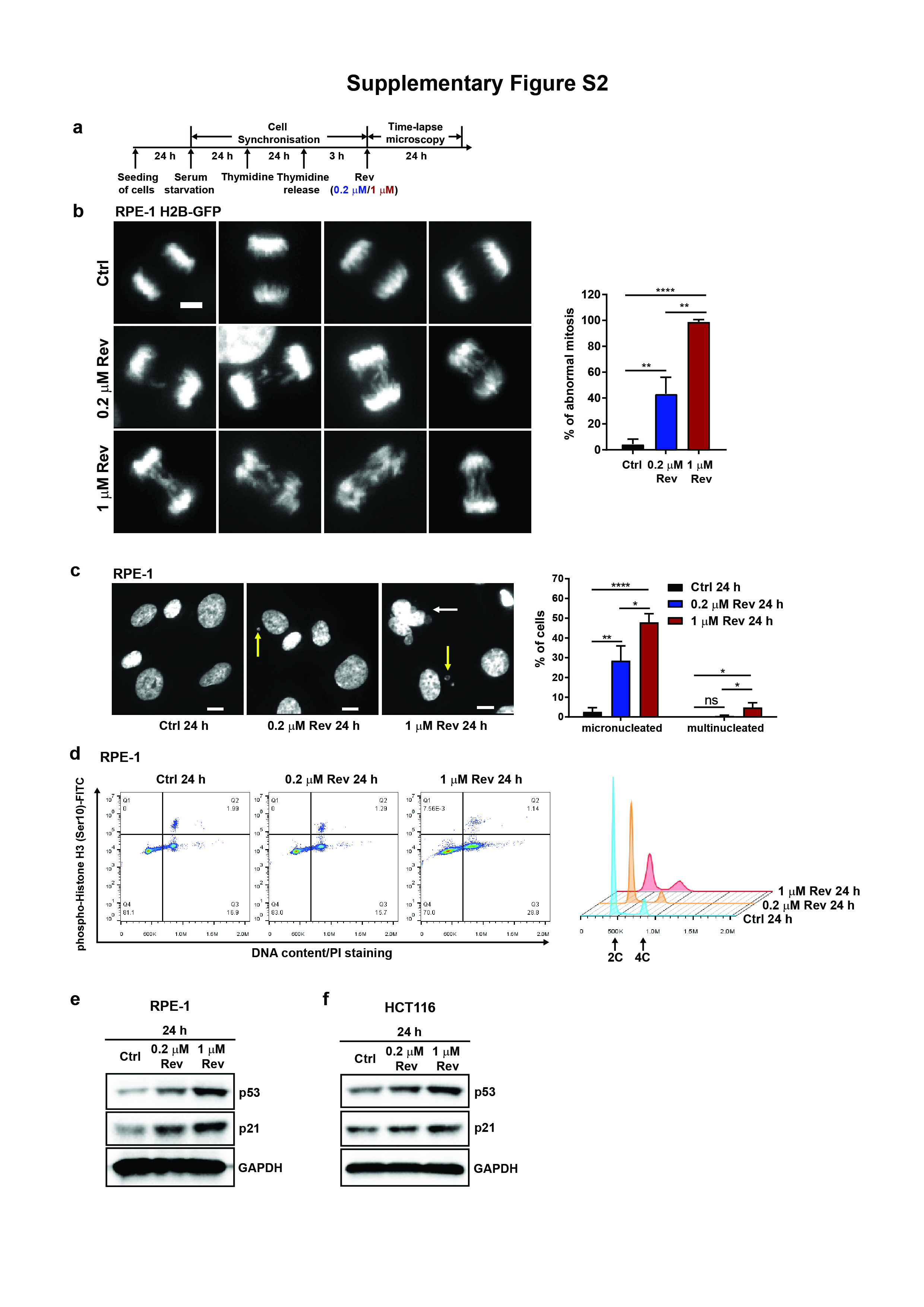

Supplement: Supplementary file 4 — Supplementary Figure S2 [file 41389_2018_72_MOESM4_ESM.jpg]

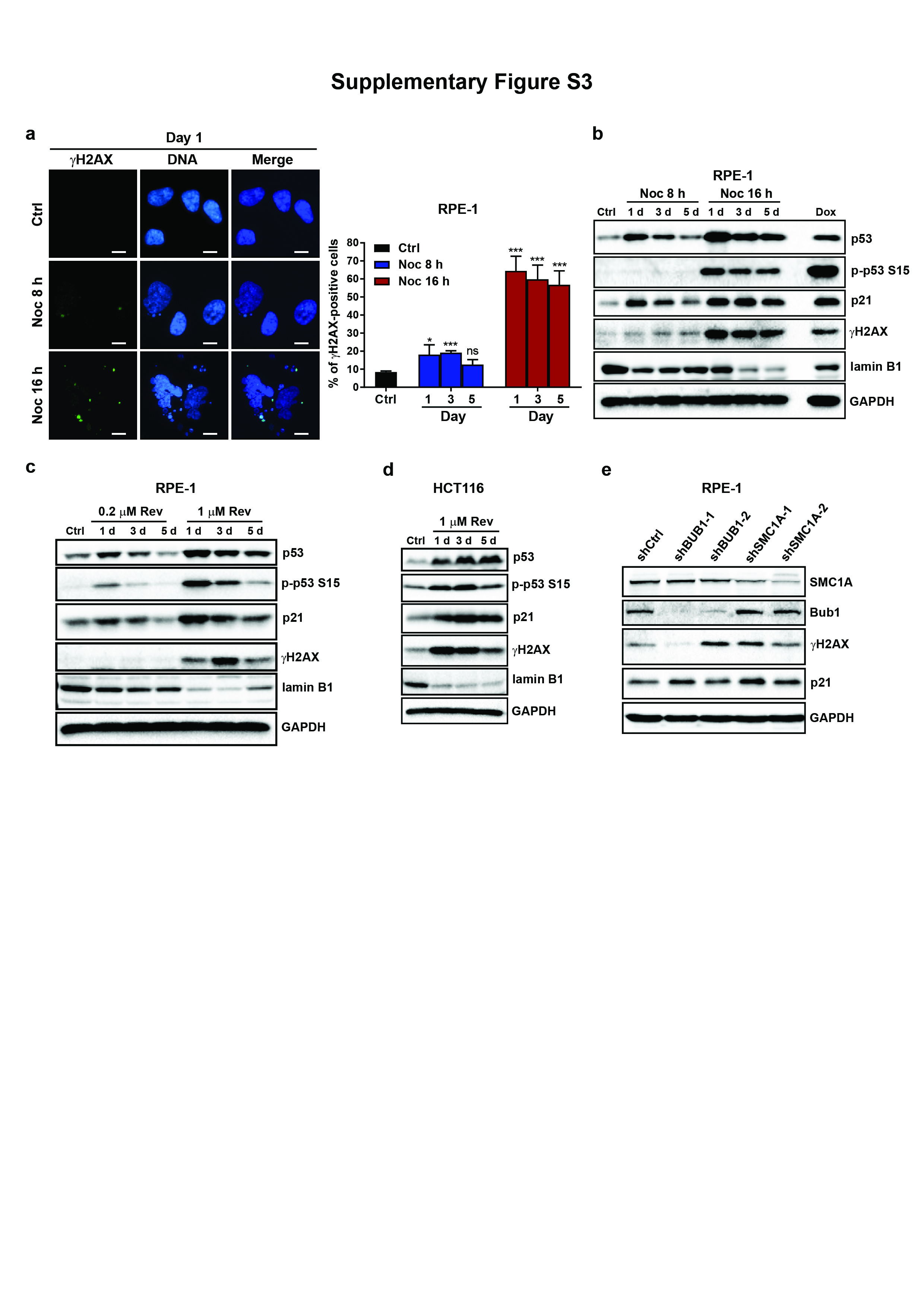

Supplement: Supplementary file 5 — Supplementary Figure S3 [file 41389_2018_72_MOESM5_ESM.jpg]

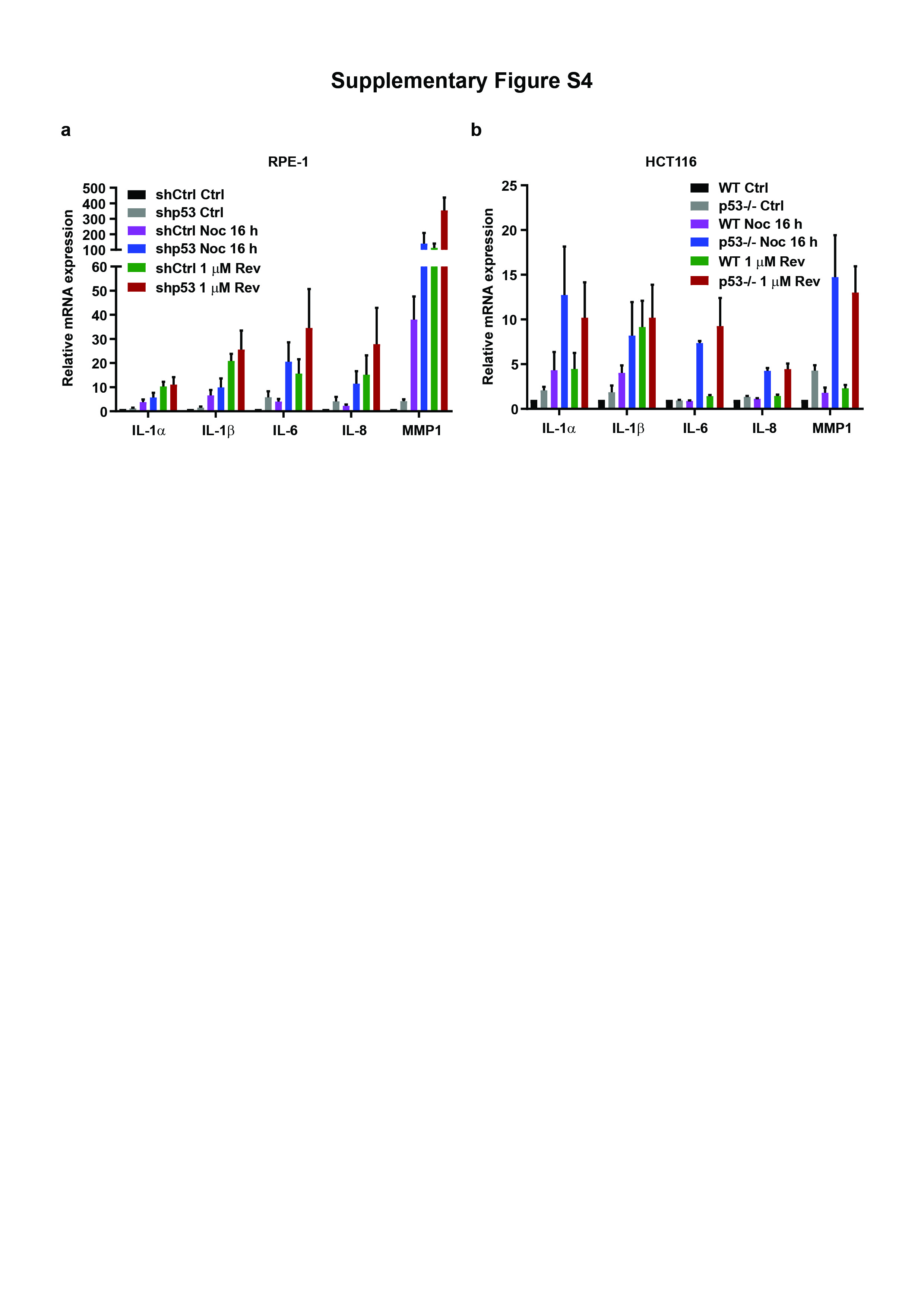

Supplement: Supplementary file 6 — Supplementary Figure S4 [file 41389_2018_72_MOESM6_ESM.jpg]

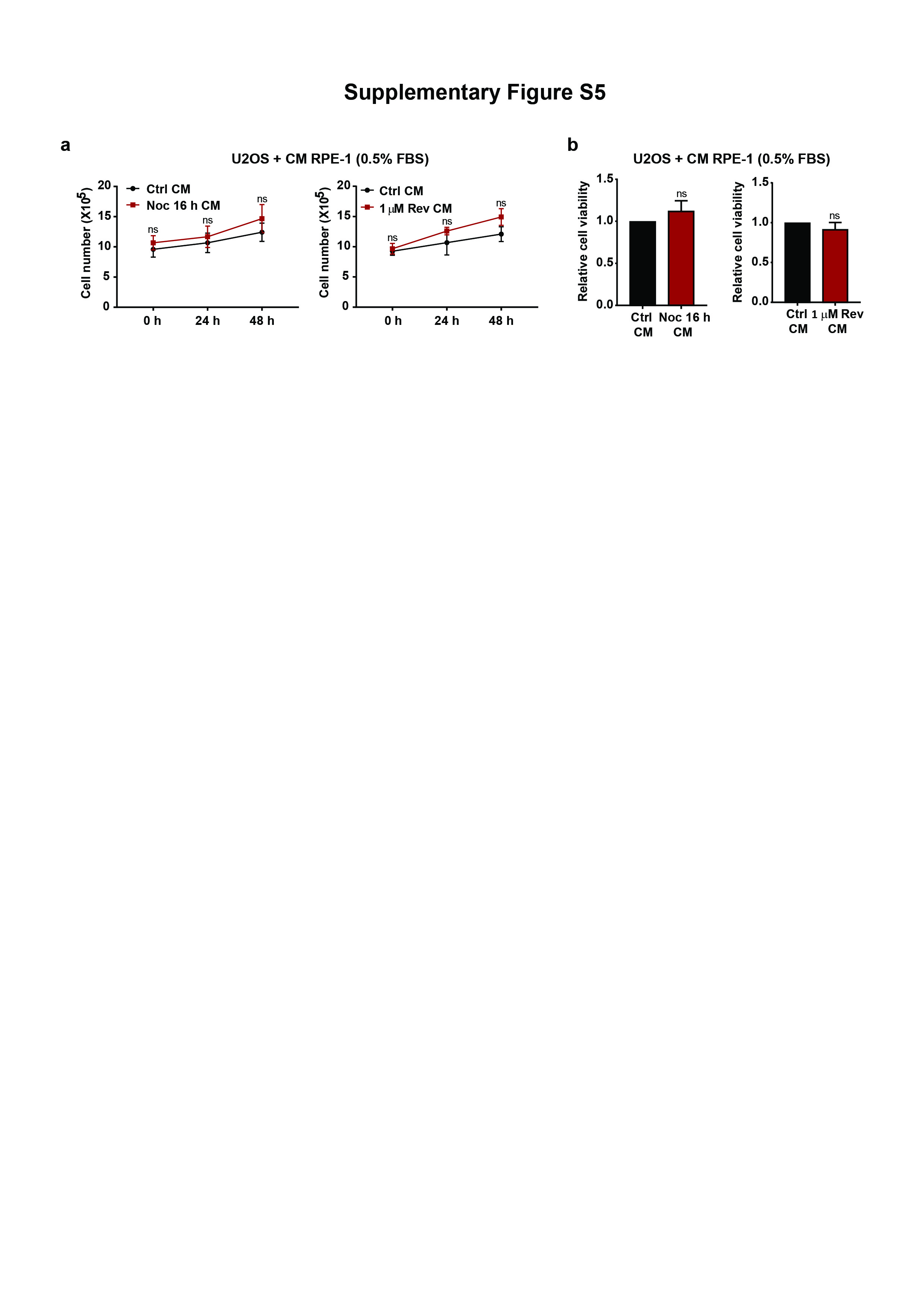

Supplement: Supplementary file 7 — Supplementary Figure S5 [file 41389_2018_72_MOESM7_ESM.jpg]

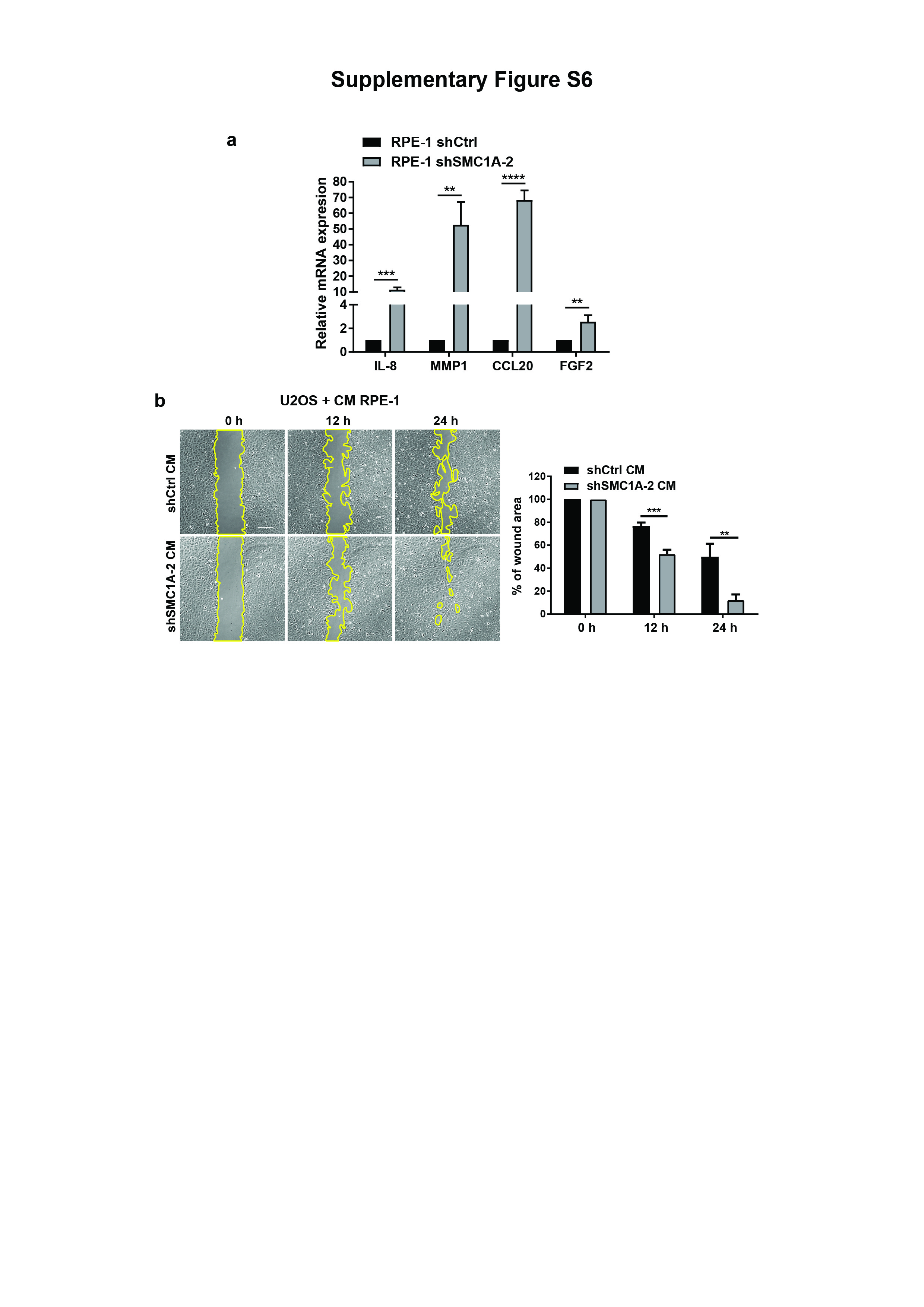

Supplement: Supplementary file 8 — Supplementary Figure S6 [file 41389_2018_72_MOESM8_ESM.jpg]

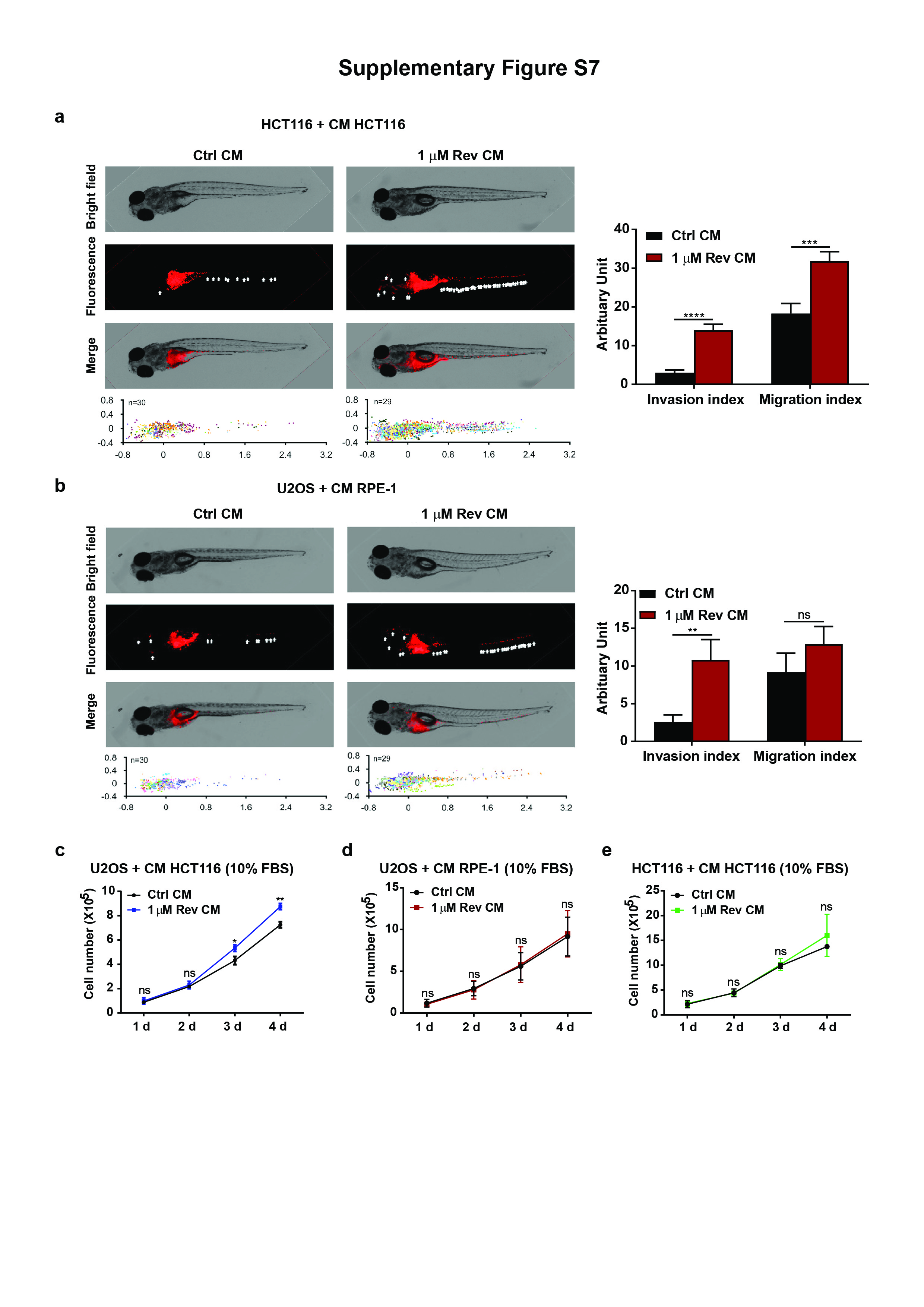

Supplement: Supplementary file 9 — Supplementary Figure S7 [file 41389_2018_72_MOESM9_ESM.jpg]

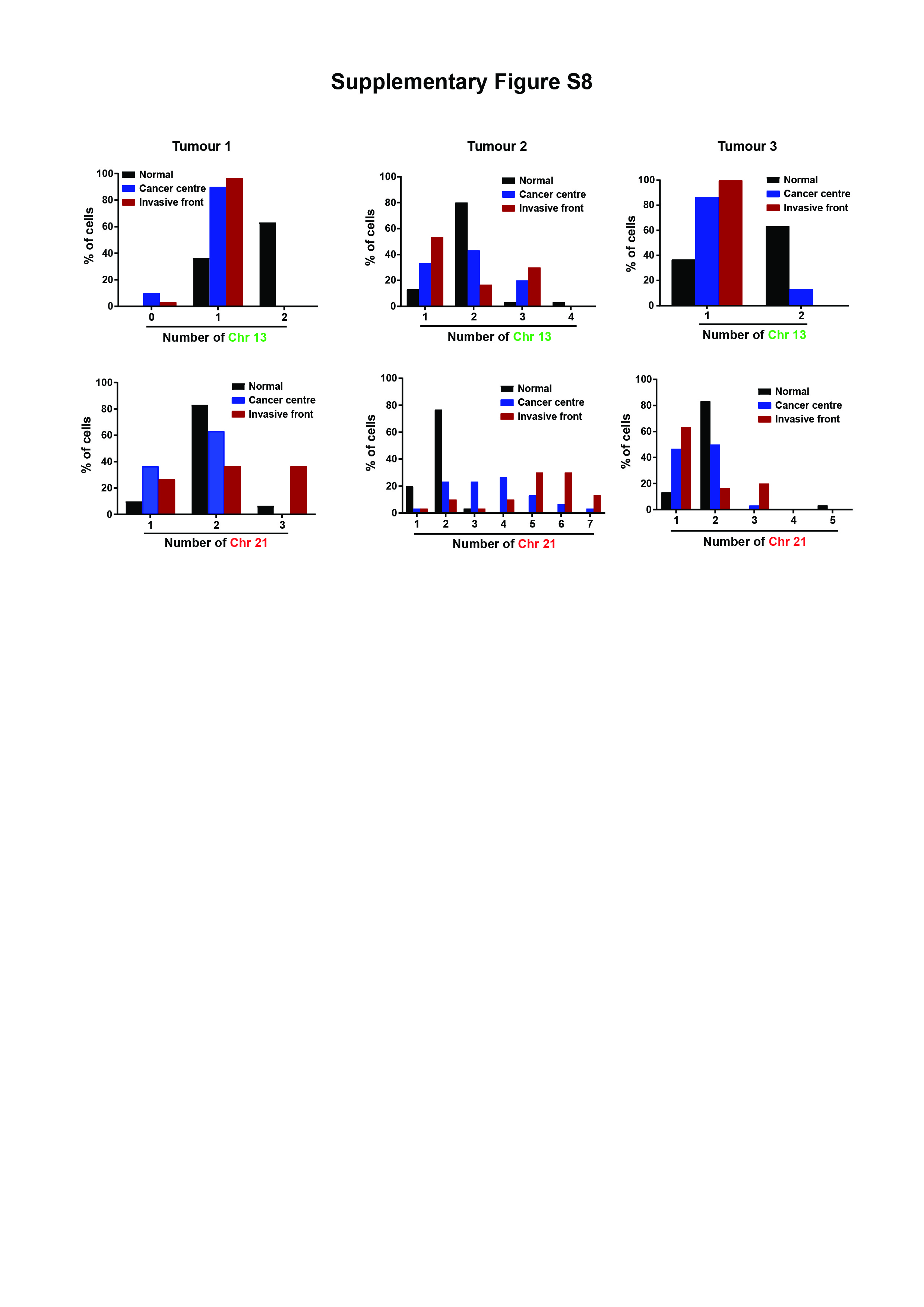

Supplement: Supplementary file 10 — Supplementary Figure S8 [file 41389_2018_72_MOESM10_ESM.jpg]
