# Supplementary material for: Chromosomal instability-induced senescence potentiates cell non-autonomous tumourigenic effects
Source: Oncogenesis. 2018 Aug 15;7(8):62. doi: 10.1038/s41389-018-0072-4 (PMC6092349; doi:10.1038/s41389-018-0072-4)
Supplement: Supplementary file 14 — Supplementary Table S4-S8 [file 41389_2018_72_MOESM14_ESM.docx]

**Supplementary Table S4. shRNA plasmids used in this study.**

| Target gene | Company | Catalog number |
| --- | --- | --- |
| shp53 | Addgene | #19119 |
| shBUB1-1 | Dharmacon | TRCN0000040157 |
| shBUB1-2 | Dharmacon | TRCN0000010307 |
| shSMC1A-1 | Dharmacon | TRCN0000062555 |
| shSMC1A-2 | Dharmacon | TRCN0000062557 |

## Supplementary Table S5. List of primers for qRT-PCR in cell lines.

| **Gene** | **Forward primer (5' to 3')** | **Reverse primer (5' to 3')** |
| --- | --- | --- |
| IL-1α | GCCCAAGATGAAGACCAACC | AGTCAGTGATAGAGGGTGGC |
| IL-1β | ACAGATGAAGTGCTCCTTCCA | GTCGGAGATTCGTAGCTGGAT |
| IL-6 | CCAGGAGCCCAGCTATGAAC | CCCAGGGAGAAGGCAACTG |
| IL-8 | GAGTGGACCACACTGCGCCA | TCCACAACCCTCTGCACCCAGT |
| COX-2 | CCTGAGCATCTACGGTTTGC | GAACAACTGCTCATCACCCC |
| BMP2 | CAAGCCAAACACAAACAGCG | CCAACGTCTGAACAATGGCA |
| GDF15 | TTGCACTCCGAAGACTCCAG | CGCACTTCTGGCGTGAGTAT |
| SPP1 | GGACAGCCAGGACTCCATTG | TGTGGGGACAACTGGAGTGAA |
| AREG | GTGGTGCTGTCGCTCTTGATA | ACTCACAGGGGAAATCTCACT |
| TGF-α | CCCAGATTCCCACACTCAGT | ACCTGGCAGCAGTGTATCAG |
| TGF-β | CCCAGCATCTGCAAAGCTC | GTCAATGTACAGCTGCCGCA |
| FGF2 | GGCTTCTTCCTGCGCATCCA | GCTCTTAGCAGACATTGGAAGA |
| S100A7 | AAAGCAAAGATGAGCAACAC | AAGTTCTCCTTCATCATCGTC |
| JAG 1 | CATCGATGAATGTGCCAGCA | GCAGTGGTCTTTCAGGTGTG |
| LRG1 | GCTGAGGGTGCTGGATCTAA | TGGTTCTCCCCAAGGTCAAG |
| ANGPTL4 | GGGTCTGGAGAAGGTGCATA | GTGGAGAAGGGTACGGAGAG |
| VEGF-A | CTTGCCTTGCTGCTCTACC | CACACAGGATGGCTTGAAG |
| MMP1 | CTACCCGGAAGTTGAGCTCA | CTAGGGAAGCCAAAGGAGCT |
| CCL20 | CTGGCTGCTTTGATGTCAGT | CGTGTGAAGCCCACAATAAA |
| PGF | CAGAGGTGGAAGTGGTACCCTTCC | CGGATCTTTAGGAGCTGCATGGTGAC |
| PDGFA | GTCCGCCAACTTCCTGATCT | CCGTGTCCTCTTCCCGATAA |
| Klf4 | TCTCCCACATGAAGCGACTT | ATGGGTCAGCGAATTGGAGA |
| Klf5 | TCCACAACAGGCCACTTACT | TGTGAGTTAACTGGCAGGGT |
| PTHLH | GCGACGATTCTTCCTTCACC | TCTTCCCAGGTGTCTTGAGC |
| WNT5A | CAGGAGTTGCTTTGGGGATG | TGGTCCTGATACAAGTGGCA |
| HMOX1 | CCCACGCCTACACCCGCTAC | GGTGGCACTGGCAATGTTGG |
| ATF3 | GGGTCACTGGTGTTTGAGGA | CCTCGGCTTTTGTGATGGAC |
| Sestrin 2 | GGCTCATCACCAAGGAACAC | CAGAGTTGTTCAACGGGTCC |
| Mieap | AGAGAATAGGCGGTCAGAGC | ACCTTCCTTGGAGTGCACTT |
| BTG2 | AGCACTACAAACACCACTGG | GTACAAGACGCAGATGGAGC |
| P8 | GTCGCACCAAGAGAGAAGCT | CCCCTCGCTTCTTCCTCTC |
| DDIT3 | CATTGCCTTTCTCCTTCGGG | CCAGAGAAGCAGGGTCAAGA |
| TNFRSF10A | AAGCTGAAGGGTCTCAGAGG | CTGTACCAGCTCTGACCACA |
| TNFRSF10B | ACCCAACAAGACCTAGCTCC | CTGTGTTTCTGGTCGTGGTG |
| Fas | TGCCCAAGTGACTGACATCA | CATCCCCATTGACTGTGCAG |
| NOXA | GCAAGAACGCTCAACCGA | TGCCGGAAGTTCAGTTTGTC |
| ARL6IP1 | TGCTTGGCTGACTACCTTGT | TGAGAAGCAGGTTGTGGACT |
| PIM1 | AAGAAGGTGAGCTCGGGTTT | TTTTCGTCCTTGATGTCGCG |
| FoxM1 | GGAGGAAATGCCACACTTAGCG | TAGGACTTCTTGGGTCTTGGGGTG |
| EZH2 | GGGACAGTAAAAATGTGTCCTGC | TGCCAGCAATAGATGCTTTTTG |
| DEK | TGTTAAGAAAGCAGATAGCAGCACC | ATTAAAGGTTCATCATCTGAACTATCCTC |
| lamin B1 | CACTGGCGAAGATGTGAAGG | CCCTGCTGGTGGAAAAGTTC |
| WHSC1 | AATATGACTCCTTGCTGGAGCAGG | ATTTCAACAGGTGGTCTTTGTCTC |
| DNMT1 | CCATCAGGCATTCTACCA | CGTTCTCCTTGTCTTCTCT |
| HMGB1 | TTGTGCAAACTTGTCGGGAG | CTCTTGGGTGCATTGGGATC |
| ITGA2 | CAGCTTCTCACCTGCAACTC | TGTGTCTTTGTGGGGCCTAT |
| Nectin 4 | ACATTGGCAGAGAAGGAGCT | TGCTTCCCAGAGTCTTCCTG |
| PMEPA1 | GAGAAGATGCCCTGTCCTCA | CTGCAGGTACGGATAGGTGG |
| L1CAM | CATCTACGTTGTCCAGCTGC | CGTCCTGAAGCACTGTTGTC |
| INHBA | TCGCACAGACCTTTCCTCAT | ATCCAGTCATTCCAGCCGAT |
| β-actin | AGAGCTACGAGCTGCCTGAC | AGCACTGTGTTGGCGTACAG |
| GAPDH | GGTCGTATTGGGCGCCTGGTCACC | CACACCCATGACGAACATGGGGGC |

## Supplementary Table S6. List of primers for qRT-PCR in human breast tumours.

| **Gene** | **Forward primer (5' to 3')** | **Reverse primer (5' to 3')** |
| --- | --- | --- |
| MMP2 | CTCAGATCCGTGGTGAGATCT | CTTTGGTTCTCCAGCTTCAGG |
| MMP9 | ATCCAGTTTGGTGTCGCGGAGC | GAAGGGGAAGACGCACAGCT |
| CCL2 | CAGCCAGATGCAATCAATGCC | TGGAATCCTGAACCCACTTCT |
| CCL5 | ATCCTCATTGCTACTGCCCTC | GCCACTGGTGTAGAAATACTCC |
| IL-6 | GGTACATCCTCGACGGCATCT | GTGCCTCTTTGCTGCTTTCAC |
| IL-8 | ATGACTTCCAAGCTGGCCGTGGCT | TCTCAGCCCTCTTCAAAACTTCTC |
| β-actin | CATGTACGTTGCTATCCAGGC | CTCCTTAATGTCACGCACGAT |

**Supplementary Table S7. Primary antibodies used in this study.**

| Antibody | Company | Catalog number |
| --- | --- | --- |
| p53 | Santa Cruz Biotechnology | sc-126 |
| phospho-p53 (Ser15) | Cell Signaling Technology | #9284 |
| phospho-Histone H2AX (Ser139) | Cell Signaling Technology | #2577 |
| p21 | Cell Signaling Technology | #2947 |
| lamin B1 | Abcam | ab16048 |
| Bub1 | Bethyl Laboratories | A300-373A |
| SMC1A | Bethyl Laboratories | A300-055A |
| Rb | Cell Signaling Technology | #9309 |
| phospho-Rb (Ser780) | Cell Signaling Technology | #9307 |
| p27 | Abcam | ab193379 |
| GAPDH | Merck Millipore | CB1001 |
| actin | Cell Signaling Technology | #4967 |

**Supplementary Table S8. Quantitative H scoring method.**

| Intensity of immunostaining (I) | 0 | | | 1 | | | | 2 | | | | 3 | | |
| --- | --- | --- | --- | --- | --- | --- | --- | --- | --- | --- | --- | --- | --- | --- |
|  | negative | | | weak | | | | moderate | | | | Strong | | |
| Percentage of stained cells in each intensity (P) | 0 | 10 | 20 | | 30 | 40 | 50 | | 60 | 70 | 80 | | 90 | 100 |
|  | 0% | 0-10% | 11-20% | | 21-30% | 31-40% | 41-50% | | 51-60% | 61-70% | 71-80% | | 81-90% | 91-100% |

H score was determined by summing the product of P (1-3) and percentage of stained cells in each intensity (0-100); Maximum = 300.
